# Six New Vibralactone Derivatives from Cultures of the Fungus *Boreostereum vibrans*

**DOI:** 10.1007/s13659-014-0029-z

**Published:** 2014-07-22

**Authors:** He-Ping Chen, Zhen-Zhu Zhao, Rong-Hua Yin, Xia Yin, Tao Feng, Zheng-Hui Li, Kun Wei, Ji-Kai Liu

**Affiliations:** 1 State Key Laboratory of Phytochemistry and Plant Resources in West China, Kunming Institute of Botany, Chinese Academy of Sciences, Kunming, 650201 People’s Republic of China; 2 University of Chinese Academy of Sciences, Beijing, 100049 People’s Republic of China

**Keywords:** *Boreostereum vibrans*, Cultural broth, Vibralactone derivatives

## Abstract

Phytochemical reinvestigation on the cultural broth of *Boreostereum vibrans* led to the isolation of six new vibralactone derivatives, vibralactone N (**1**), vibralactone O (**2**), vibralactone P (**3**), 10-lactyl vibralactone G (**4**), (3S*, 4R*)-6-acetoxymethyl-2,2-dimethyl-3,4-dihydro-2*H*-chromene-3,4-diol (**5**), vibralactone Q (**6**). Their structures were elucidated by extensive spectroscopic methods.

.

**Electronic supplementary material:**

The online version of this article (doi:10.1007/s13659-014-0029-z) contains supplementary material, which is available to authorized users.

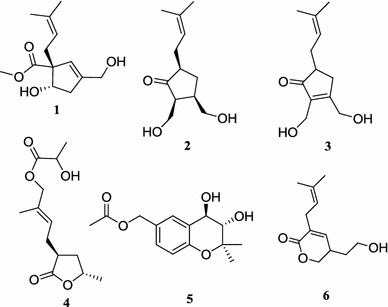

## Introduction

*Boreostereum vibrans* (synonym *Stereum vibrans*) is a fungus belonged to the family Boreostereaceae which is characterized by possessing diverse bioactive compounds [1–7]. Vibralactone, first reported in 2006, is a rare fused *β*-lactone isolated from *B. vibrans* with significant lipase inhibitory activity (IC_50_ = 0.4 μg/mL) [8]. This distinguished compound has aroused many follow-up studies. In 2008, first total synthesis of vibralactone was reported [9, 10]. In 2011, vibralactone was used as a tool to study the activity and structure of the ClpP1P2 complex from *Listeria monocytogenes* was published [11]. Our continuous investigations on the chemical constituents of the culture of *B. vibrans* have led to a series of reports on bioactive vibralactone derivatives [12–15]. Recently the biosynthetic origin of vibralactone and its biosynthetic pathway which includes several very interesting reactions were established [16]. In order to explore and understand the potential for the production of secondary metabolites by *B. vibrans*, a scale-up fermentation of this fungus was carried out. Very careful investigation of the culture has resulted in the isolation of six new vibralactone derivatives. This paper deals with the isolation and structure elucidation of these compounds.

## Results and Discussion

**Fig. 1:**
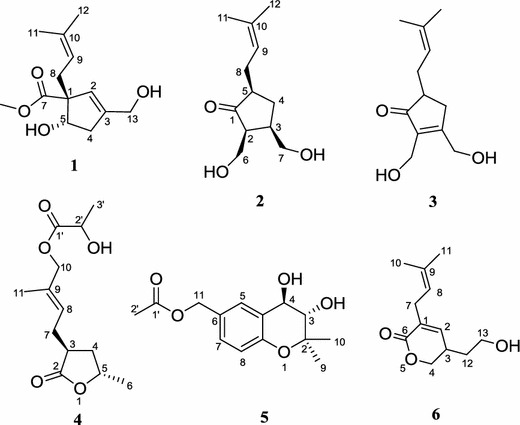
Structures of compounds **1**–**6**

Compound **1** was isolated as a colorless oil and determined to have a molecular formula of C_13_H_20_O_4_ based on the HREIMS data, *m/z* 240.1361 [M]^+^ (calcd for 240.1362). The IR absorption bands at 3430, 1729 and 1634 cm^−1^ suggested the presence of hydroxyl, carboxyl and double bond groups, respectively. From its ^1^H NMR spectrum, two olefinic protons (*δ*_H_ 5.06 and 5.61) can obviously be found, as well as three methyl groups at *δ* 1.57 (3H, s), 1.65 (3H, s) and 3.61 (3H, s). The ^13^C NMR and DEPT spectra of **1** showed thirteen carbons resonances, including three methyls, three methylenes, four olefinic carbons, an oxymethine, and two quaternary carbons (*δ*_C_ 65.8, C-1; 174.5, C-7; Table 1). Detailed analyses of the 2D NMR spectra of **1** revealed that it was similar to those of vibralactone E [12]. In the HMBC spectrum of **1**, an obvious correlation was observed from a methoxy (*δ*_H_ 3.61) to the carboxyl group (*δ*_C_ 174.5, C-7). To draw a conclusion, compound **1** was esterified by a methyl at the carboxyl group of vibralactone E. The relative configuration of **1** was determined by a ROESY experiment. In the ROESY spectrum, H-5 correlated to the two protons of C-8, indicating that both H-5 and the isopentenyl group were *β* oriented. Compound **1** was named vibralactone N (Fig. 1).

**Table 1:** ^1^H NMR (600 MHz) and ^13^C NMR (150 MHz) data of compounds **1–3** (*δ* in ppm, *J* in Hz)

Position	1^a^		2^a^		3^b^	
	*δ*_C_, type	*δ*_H_, multi.	*δ*_C_, type	*δ*_H_, multi.	*δ*_C_, type	*δ*_H_, multi.
1	65.8, s		218.7, s		210.7, s	
2	125.8, d	5.61, s	57.3, d	2.04, overlapped	137.8, s	
3	145.4, s		42.6, d	2.24, m	173.2, s	
4	41.4, t	2.28, dd (16.3, 1.5)	31.0, t	1.25, m	34.1, t	2.69, dd (18.6, 6.3)
		2.67, dd (16.3, 5.6)		2.17, overlapped		2.24, dd (18.6, 1.8)
5	78.9, d	4.20, ddd (6.4, 5.6, 1.5)	50.8, d	2.16, overlapped	45.4, d	2.46, overlapped
6			61.7, t	3.81, ddd (10.5, 6.1, 4.5)	55.4, t	4.45, s, 2H
				3.64, ddd (10.5, 6.6, 6.0)		
7	174.5, s		65.8, t	3.73, ddd (11.4, 7.6, 5.8)	62.6, t	4.58, s, 2H
				3.58, ddd (11.4, 7.0, 4.4)		
8	35.8, t	2.15, dd (13.9, 7.5)	28.6, t	2.03, overlapped	29.6, t	2.48, overlapped
		2.54, dd (13.9, 7.5)		2.39, m		2.11, m
9	120.8, d	5.06, br. t (7.5)	122.7, d	5.11, br. t (7.8)	120.7, d	5.04, br. t (7.6)
10	134.3, s		133.3, s		134.3, s	
11	17.9, q	1.57, s, 3H	17.9, q	1.59, s, 3H	18.1, q	1.61, s, 3H
12	26.0, q	1.65, s, 3H	26.0, q	1.66, s, 3H	25.9, q	1.69, s, 3H
13	61.7, t	4.09, d (5.7), 2H				
7-OCH_3_	51.5, q	3.61, s, 3H				
5-OH		3.99, d (6.4)				
6-OH				4.26, dd (6.0, 4.5)		
7-OH				4.32, dd (5.8, 4.4)		
13-OH		3.85, t (5.7)				

^a^Recorded in acetone-*d*_6_

^b^Recorded in chloroform-*d*

**Fig. 2:**
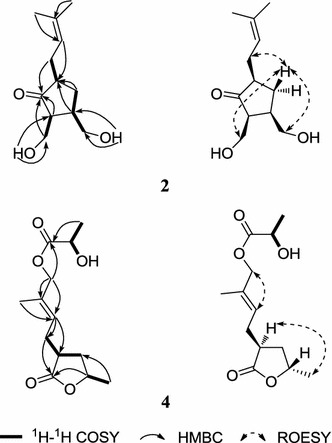
Selected 2D NMR correlations of compounds **2** and **4**

Compound **2**, a colorless oil, was determined to have a molecular formula of C_12_H_20_O_3_ according to the HREIMS data, *m/z* 212.1404 [M]^+^ (calcd for 212.1412). The IR spectrum revealed the presence of hydroxyl (3388 cm^−1^) and carboxyl (1735 cm^−1^) groups. The 1D NMR spectra demonstrated twelve carbons, which were ascribed to two methyls, four methylenes, four methines and two quaternary carbons (Table 1). These spectroscopic data showed that **2** was very similar to those of vibralactone I [14]. The analyses of ^1^H-^1^H COSY and HMBC spectra of **2** suggested that **2** possessed a same planar structure with that of vibralactone I. Its ^13^C NMR and DEPT spectra showed that the chemical shifts of C-4 (*δ*_C_ 31.0) and C-5 (*δ*_C_ 50.8) of **2** were downfield shifted, while C-1 (*δ*_C_ 218.7) was upfield shifted obviously comparing to the corresponding signals of vibralactone I (C-1, *δ*_C_ 220.2, C-4, *δ*_C_ 28.3; C-5, *δ*_C_ 47.3). These data suggested that **2** was a stereoisomer of vibralactone I. In the ROESY spectrum, a key correlation of H-4 with H-8 and strong correlations from H-8 to H-6 and H-7 suggested H-2, H-3 and H-5 were in the same side (Fig. 2). Therefore, the structure of **2** was elucidated and named vibralactone O (Fig. 1).

The molecular formula of compound **3** was established as C_12_H_18_O_3_ by HREIMS (*m/z* 210.1250, calcd for 210.1256). Compared its ^13^C NMR and DEPT spectroscopic data with those of compound **2**, the chemical shifts of the carbons in **3** were similar to the corresponding carbons in **2**, with the exception of chemical shifts of C-2 (*δ*_C_ 137.8) and C-3 (*δ*_C_ 173.2). Subsequent analysis of the 2D NMR spectroscopic data of **3** suggested the existence of an *α*,*β*-unsaturated ketone, owing to C-2 and C-3 were oxidized to form a double bond which conjugated with the carbonyl group (*δ*_C_ 210.7). The stereo-configuration of C-5 was not determined currently. Compound **3** was named vibralactone P (Fig. 1).

Compound **4** was obtained as a colorless oil. Its molecular formula was determined as C_13_H_20_O_5_ by HRESIMS (*m/z* 279.1203 [M + Na]^+^, calcd for 279.1208), with four degrees of unsaturation. The IR spectrum revealed the existence of hydroxy (3437 cm^−1^) and carboxyl (1766 cm^−1^) groups. The ^13^C NMR and DEPT spectra showed thirteen carbons, including three methyls, three methylenes, four methines (three were oxygenated) and three quaternary carbons (two olefinic carbons and two lactone carbons; Table 2). In the HMBC spectrum, the proton at 1.34 ppm (d, *J* = 6.6 Hz, H-3′) was correlated to a methine (*δ*_C_ 67.5, C-2′) and a carbonyl group (*δ*_C_ 175.4, C-1′), as well as cross peaks from 2′-OH (*δ*_H_ 4.24) and H-3′ to H-2′ (*δ*_H_ 4.25) in the COSY spectrum, revealed that the presence of a lactic acid group. Further analyses of the 2D NMR spectroscopic data of **4** suggested that the other parts of **4** were similar to those of vibralactone G both in planar structure and stereo-configuration [14]. From the HMBC spectrum, significant correlations were observed from H-10 (*δ*_H_ 4.51, dd, *J* = 12.4, 2.8 Hz; 4.55, dd, *J* = 12.4, 2.8 Hz) to the lactic carbonyl group (*δ*_C_ 175.4, C-1′). These signals confirmed that **4** was a lactyl-substituted derivative of vibralactone G. The relative configuration was determined by a ROESY experiment. The correlations from H-3 (*δ*_H_ 2.79, m) to H-6 (*δ*_H_ 1.31, d, *J* = 6.4 Hz) were found in the ROESY spectrum. We have not observed the cross peaks from H-3 to H-5 (*δ*_H_ 4.65, m) in the ROESY. It suggested that H-3 and Me-6 (*δ*_C_ 21.3) were in the same side. On the other hand, strong cross peaks were observed from H-8 (5.53, br. t, *J* = 7.8 Hz) to H-10 (4.51, dd, *J* = 12.4, 2.8 Hz; 4.55, dd, *J* = 12.4, 2.8 Hz). These evidences suggested that the double bond (C8–C9) was a *E* configuration (Fig. 2). Therefore, compound **4** was identified as 10-lactyl vibralactone G, as shown in Fig. 1.

**Table 2:** ^1^H NMR (600 MHz) and ^13^C NMR (150 MHz) data of compounds **4**–**6** (*δ* in ppm, *J* in Hz)

Position	4^a^		5^a^		6^a^	
	*δ*_C_, type	*δ*_H_, multi.	*δ*_C_, type	*δ*_H_, multi.	*δ*_C_, type	*δ*_H_, multi.
1					131.2, s	
2	178.7, s		79.5, s		144.7, d	6.67, d (3.9)
3	39.7, d	2.79, m	76.5, d	3.53, dd (8.6,6.1)	32.4, d	2.72, m
4	34.7, t	2.12, m 2.03, overlapped	69.6, d	4.51, dd (8.4,4.6)	71.0, t	4.37, dd (11.0,4.8)
						4.11, dd (11.0,7.2)
4a			125.9, s			
5	75.4, d	4.65, m	129.5, d	7.48, d (2.1)		
6	21.3, q	1.31, d (6.4), 3H	129.0, s		165.1, s	
7	29.0, t	2.46, m	130.0, d	7.16, dd (8.3,2.1)	29.9, t	2.92, overlapped, 2H
		2.33, m				
8	125.9, d	5.53, br. t (7.8)	117.2, d	6.70, d (8.3)	121.5, d	5.17, br. t (7.2)
8a			153.3, s			
9	133.7, s		19.5, q	1.18, s, 3H	134.3, s	
10	70.2, t	4.55, dd (12.4,2.8)	27.2, q	1.42, s, 3H	17.7, q	1.62, s, 3H
		4.51, dd (12.4,2.8)				
11	14.0, q	1.69, s, 3H	27.2, t	4.99, s, 2H	25.8, q	1.70, s, 3H
12					34.3, t	1.65, m, 2H
13					59.8, t	3.66, m, 2H
1′	175.4, s		170.9, s			
2′	67.5, d	4.25, overlapped	20.9, q	2.01, s, 3H		
3′	20.8, q	1.34, d (6.6), 3H				
3-OH				4.60, d (8.6)		
4-OH				4.67, d (4.6)		
13-OH						3.79, t (4.8)
2′-OH		4.24, overlapped				

^a^Recorded in acetone-*d*_6_

Compound **5** was isolated as a pale yellow solid. It had a molecular formula as C_14_H_18_O_5_ from the HREIMS (*m/z* 266.1147 [M]^+^, calcd for 266.1154). In the ^1^H NMR spectrum, the ABX spin system observed from aromatic protons at *δ*_H_ 7.48 (1H, d, *J* = 2.1 Hz), 7.16 (1H, dd, *J* = 8.3, 2.1 Hz), and 6.70 (1H, d, *J* = 8.3 Hz) reveals the presence of a 1,2,4-trisubstituted benzene ring. Besides, in the ^1^H-^1^H COSY spectrum, cross peaks from two hydroxyl protons at *δ*_H_ 4.60 (1H, d, *J* = 6.1 Hz) and 4.67 (1H, d, *J* = 4.6 Hz) to H-3 (*δ*_H_ 3.53, dd, *J* = 8.4, 6.1 Hz) and H-4 (*δ*_H_ 4.51, dd, *J* = 8.4, 4.6 Hz), respectively, as well as cross peaks from H-3 to H-4 were observed, these data suggested the existence of a 1,2-diols group. The coupling constant of H-3/4 (*J* = 8.4 Hz) suggested that the two hydroxyl groups were in the opposite side. The HMBC spectrum showed that H-11 (*δ*_H_ 4.99, s, 2H) exhibited a clear correlation to the carboxyl group (*δ*_C_ 170.9, C-1′). Meanwhile, only one signal could be detected from the methyl singlet at *δ*_H_ 2.01 ppm to the carbonyl group. All these evidences suggested that **5** was an acetylation derivative of (3S*,4R*)-6-(hydroxymethyl)-2,2-dimethyl-3,4-dihydro-2*H*-chromene-3,4-diol at the position of hydromethyl group. (3S*,4R*)-6-(Hydroxymethyl)-2,2-dimethyl-3,4-dihydro-2*H*-chromene-3,4-diol was isolated from the fermentation broth of a marine sediment-derived fungus *Eutypella scoparia* FS26 obtained from the South China Sea as one of the two new polyketides [17]. Thus, compound **5** was established as (3S*,4R*)-6-acetoxymethyl-2,2-dimethyl-3,4-dihydro-2*H*-chromene-3,4-diol, as shown in Fig. 1.

Compound **6**, a colorless oil, was determined to have a molecular formula of C_12_H_18_O_3_ based on the HREIMS data, *m/z* 210.1259 [M]^+^ (calcd for 210.1256). The strong adsorption bands at 3427 and 1717 cm^−1^ suggested the presence of hydroxyl and carboxyl groups. The 1D NMR spectroscopic data demonstrated twelve carbons signals, including four olefinic carbons and a carbonyl. According to the HMBC spectrum, correlations can be found from both two methyl singlets (*δ*_H_1.62, H-10; 1.70, H-11) to two olefinic carbons (C-8, C-9). Meanwhile, cross peaks from H-7 to H-8 were also displayed in the ^1^H-^1^H COSY spectrum. These data confirmed the presence of an isopentenyl unit. Furthermore, the ^1^H-^1^H COSY correlations established connections from C-2/C-3/C-12/C-13. The HMBC spectrum showed that H-7 (*δ*_H_ 2.92) correlated to C-1, C-2 and C-6, H-2 (*δ*_H_ 6.67, d, *J* = 3.9 Hz) correlated to C-4 and C-6, as well as H-4 (*δ*_H_ 4.11, 1H, dd, *J* = 10.8, 7.2 Hz; 4.37, 1H, dd, *J* = 10.8, 4.8 Hz) correlated to C-6. The stereo-configuration of C-3 was not determined currently. Compound **6** was named vibralactone Q (Fig. 1).

## Experimental Section

### General Experimental Procedures

UV spectra were obtained using a Shamashim UV 2401 spectrometer. Optical rotations were recorded on a JASCO P-1020 polarimeter. IR spectra were measured on a Bruker Tensor-27 infrared spectrophotometer with KBr pellets. HREIMS were obtained on a Waters Autospec Premier P776 mass spectrometer. HRESIMS were taken on an Agilent G6230 TOF MS spectrometer. 1D and 2D NMR spectra were recorded on Bruker Avance-600 and Ultrashield-800 spectrometers using TMS as an internal standard. Silica gel 200–300 mesh (Qingdao Marine Chemical Inc., China) and Sephadex LH-20 (Amersham Biosciences, Sweden) were used for column chromatography. Medium pressure liquid chromatography (MPLC) was performed on a Büchi Sepacore System equipping pump manager C-615, pump modules C-605 and fraction collector C-660 (Büchi Labortechnik AG, Switzerland), and columns packed with Chromatorex C-18 (40–75 μm, Fuji Silysia Chemical Ltd., Japan). Preparative HPLC was performed on an Agilent 1260 liquid chromatography system equipped with a Zorbax SB-C18 column (5 μm, 9.4 mm × 150 mm).

### Fungus Material and Cultivation Conditions

The fungus *B. vibrans* was provided and fermented by Zheng-Hui Li, Kunming Institute of Botany, Chinese Academy of Sciences. A voucher specimen (No. 20120920B) was deposited at the Herbarium of Kunming Institute of Botany. The culture medium to ferment this fungus consist of glucose (5 %), peptone from porcine meat (0.15 %), yeast powder (0.5 %), KH_2_PO_4_ (0.05 %) and MgSO_4_ (0.05 %). Five hundred 500-mL Erlenmeyer flasks each containing 350 mL of above-mentioned culture medium were inoculated with *B. vibrans* strains, respectively. Then they were incubated on rotary shakers at 24 °C and 150 rpm for 25 days in dark environment.

### Extraction and Isolation

The culture broth (400 L) of *B. vibrans* was filtered, and the filtrate was extracted four times with ethyl acetate (EtOAc). Meanwhile, the mycelium was extracted by CHCl_3_/MeOH (1:1) for three times. The EtOAc layer together with the mycelium extraction was concentrated under reduced pressure to afford a crude extract (353 g). Then this residue was subjected to column chromatography over silica gel (200–300 mesh) eluting with a gradient of petroleum ether/acetone (100:0 → 0:100) to give five fractions (A–E). Fraction B was separated by MPLC eluting with (MeOH/H_2_O, 10:90 → 100:0) to afford ten subfractions (B1–B10). Subfraction B4 was subjected to normal phase column chromatography eluting with petroleum ether/acetone (4:1 → 2:1) to give **3** (0.5 mg). Subfraction B5 was subjected to reverse phase column chromatography eluting with MeOH/H_2_O (40:60 → 60:40) followed by Sephadex LH-20 (acetone) column chromatography to yield **1** (3.5 mg) and **2** (1.8 mg). Subfraction B7 was separated by preparative HPLC (MeCN/H_2_O, 15/85, 10 mL/min), and then purified by Sephadex LH-20 (acetone) column chromatography to afford **4** (2.3 mg) and **5** (0.8 mg). Subfraction B8 was purified by preparative HPLC (MeCN/H_2_O, 20/80, 10 mL/min) to yield **6** (3.8 mg).

### Vibralactone N (**1**)

 Colorless oil; [α]${}_{\text{D}}^{21}$+5.3 (*c* 0.12, MeOH). UV (MeOH) λ_max_ nm (log *ε*): 202 (3.03). IR (KBr) ν_max_ cm^−1^: 3430, 2964, 2924, 2857, 1729, 1634, 1439, 1384, 1233, 1057. For ^1^H (600 MHz, acetone-*d*_6_) and ^13^C NMR (150 MHz, acetone-*d*_6_) data, see Table 1. HREIMS *m/z*: 240.1361 [M]^+^ (calcd for C_13_H_20_O_4_, 240.1362).

### Vibralactone O (**2**)

 Colorless oil; [α]${}_{\text{D}}^{21}$+82.8 (*c* 0.05, MeOH). UV (MeOH) λ_max_ nm (log *ε*): 203 (3.60), 302 (1.94). IR (KBr) ν_max_ cm^−1^: 3388, 2964, 2924, 2878, 1735, 1452, 1378, 1154, 1075, 1053, 1027. For ^1^H (600 MHz, acetone-*d*_6_) and ^13^C NMR (150 MHz, acetone-*d*_6_) data, see Table 1. HREIMS *m/z*: 212.1404 [M]^+^ (calcd for C_12_H_20_O_3_, 212.1412).

### Vibralactone P (**3**)

Yellow oil; [α]${}_{\text{D}}^{21}$−24.0 (*c* 0.02, MeOH). UV (MeOH) λ_max_ nm (log *ε*): 201 (3.30), 231 (3.33), 388 (1.75). For ^1^H (600 MHz, CDCl_3_) and ^13^C NMR (150 MHz, CDCl_3_) data, see Table 1. HREIMS *m/z*: 210.1250 [M]^+^ (calcd for C_12_H_18_O_3_, 210.1256).

### 10-Lactyl vibralactone G (**4**)

Colorless oil; [α]${}_{\text{D}}^{21}$−15.6(*c* 0.05, MeOH). UV (MeOH) λ_max_ nm (log *ε*): 202 (3.74). IR (KBr) ν_max_ cm^−1^: 3437, 2979, 2935, 2879, 1766, 1632, 1454, 1385, 1199, 1132, 1042, 956. For ^1^H (600 MHz, acetone-*d*_6_) and ^13^C NMR (150 MHz, acetone-*d*_6_) data, see Table 2. HRESIMS *m/z*: 279.1203 [M + Na]^+^ (calcd for C_13_H_20_O_5_Na, 279.1208).

### (3S*,4R*)-6-Acetoxymethyl-2,2-dimethyl-3,4-dihydro-2H-chromene-3,4-diol (**5**)

Pale yellow solid; [α]${}_{\text{D}}^{21}$−30.0 (*c* 0.02, MeOH). UV (MeOH) λ_max_ nm (log *ε*): 203 (4.26), 230 (3.74), 280 (2.98). IR (KBr) ν_max_ cm^−1^: 3451, 2924, 2935, 2871, 1723, 1639, 1497, 1384, 1252, 1033. For ^1^H (600 MHz, acetone-*d*_6_) and ^13^C NMR (150 MHz, acetone-*d*_6_) data, see Table 2. HREIMS *m/z*: 266.1147 [M]^+^ (calcd for C_14_H_18_O_5_, 266.1154).

### Vibralactone Q (**6**)

 Colorless oil; [α]${}_{\text{D}}^{21}$−6.8 (*c* 0.09, MeOH). UV (MeOH) λ_max_ nm (log *ε*): 204 (3.96). IR (KBr) ν_max_ cm^−1^: 3427, 2972, 2932, 1718, 1640, 1450, 1403, 1383, 1200, 1134, 1063. For ^1^H (600 MHz, acetone-*d*_6_) and ^13^C NMR (150 MHz, acetone-*d*_6_) data, see Table 2. HREIMS *m/z*: 210.1259 [M]^+^ (calcd for C_12_H_18_O_3_, 210.1256).

## Electronic supplementary material

Below is the link to the electronic supplementary material.

Supplementary material 1 (DOCX 6801 kb)

## References

[1] Isaka M, Srisanoh U, Choowong W, Boonpratuang T (2011). Org. Lett..

[2] Isaka M, Srisanoh U, Sappan M, Supothina S, Boonpratuang T (2012). Phytochemistry.

[3] Li GH, Liu FF, Shen L, Zhu HJ, Zhang KQ (2011). J. Nat. Prod..

[4] Liermann JC, Schueffler A, Wollinsky B, Birnbacher J, Kolshorn H, Anke T, Opatz T (2010). J. Org. Chem..

[5] Liu FF, Li GH, Yang ZS, Zheng X, Yang Y, Zhang KQ (2010). Helv. Chim. Acta.

[6] Ma K, Bao L, Han JJ, Jin T, Yang XL, Zhao F, Li SF, Song FH, Liu MM, Liu HW (2014). Food Chem..

[7] Zheng X, Li GH, Xie MJ, Wang X, Sun R, Lu H, Zhang KQ (2013). Phytochemistry.

[8] Liu DZ, Wang F, Liao TG, Tang JG, Steglich W, Zhu HJ, Liu JK (2006). Org. Lett..

[9] Zhou Q, Snider BB (2008). Org. Lett..

[10] Zhou Q, Snider BB (2008). J. Org. Chem..

[11] Zeiler E, Braun N, Böttcher T, Kastenmüller A, Weinkauf S, Sieber SA (2011). Angew. Chem. Int. Ed..

[12] Jiang MY, Zhang L, Dong ZJ, Yang ZL, Leng Y, Liu JK (2010). Chem. Pharm. Bull..

[13] Ding JH, Feng T, Li ZH, Li L, Liu JK (2012). Nat. Prod. Bioprospect..

[14] Wang GQ, Wei K, Feng T, Li ZH, Zhang L, Wang QA, Liu JK (2012). J. Asian Nat. Prod. Res..

[15] Wang GQ, Wei K, Li ZH, Feng T, Ding JH, Wang QA, Liu JK (2013). J. Asian Nat. Prod. Res..

[16] Zhao PJ, Yang YL, Du L, Liu JK, Zeng Y (2013). Angew. Chem. Int. Ed..

[17] Sun L, Li DL, Tao MH, Chen YC, Zhang QB, Dan FJ, Zhang WM (2013). Nat. Prod. Res..

