# Supplementary material for: Six New Vibralactone Derivatives from Cultures of the Fungus Boreostereum vibrans
Source: Nat Prod Bioprospect. 2014 Jul 22;4(5):271–6. doi: 10.1007/s13659-014-0029-z (PMC4199947; doi:10.1007/s13659-014-0029-z)

**Electronic Supplementary Material**

**Six new vibralactone derivatives from cultures of the fungus *Boreostereum vibrans***

He-Ping Chen,^a,b^ Zhen-Zhu Zhao,^a,b^ Rong-Hua Yin,^a,b^ Xia Yin,^a,b^ Tao Feng,^a^ Zheng-Hui Li,^a^ Kun Wei^a^^,^* and Ji-Kai Liu^a,^*

^a^State Key Laboratory of Phytochemistry and Plant Resources in West China, Kunming Institute of Botany, Chinese Academy of Sciences, Kunming 650201, China

^b^University of Chinese Academy of Sciences, Beijing 100049, China

To whom correspondence should be addressed. E-mail: jkliu@mail.kib.ac.cn; weikun@mail.kib.ac.cn

**Content**

**Figure 1S-7S.** NMR and MS spectra of compound **1**

**Figure 8S-14S.** NMR and MS spectra of compound **2**

**Figure 15S-21S.** NMR and MS spectra of compound **3**

**Figure 22S-28S.** NMR and MS spectra of compound **4**

**Figure 29S-35S.** NMR and MS spectra of compound **5**

**Figure 36S-42S.** NMR and MS spectra of compound **6**

**Figure 1S.**^1^H NMR (600 MHz) spectrum of compound **1** in acetone-*d*_6_.


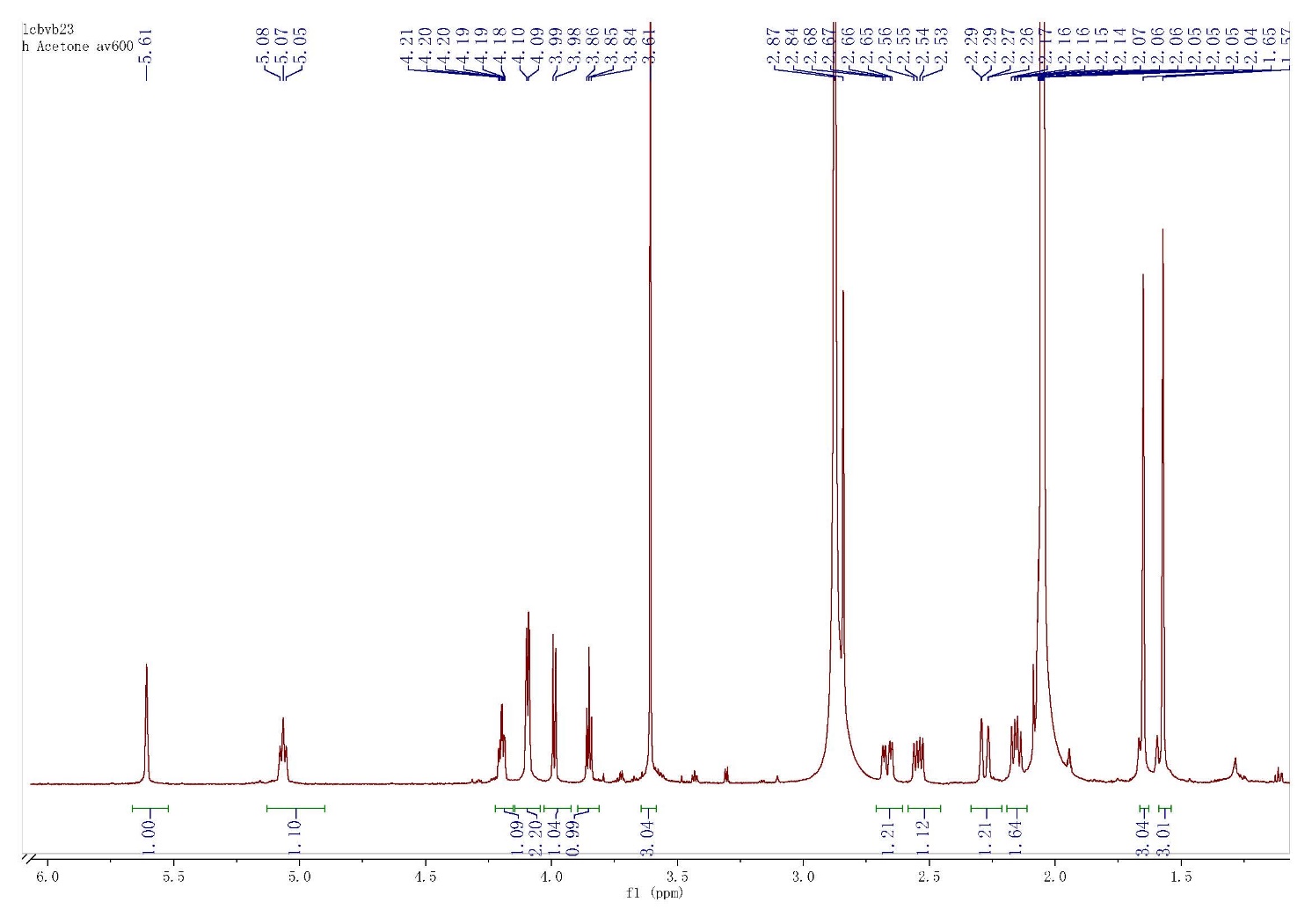


**Figure 2S.**^13^C NMR(150 MHz) and DEPT spectra of compound **1** in acetone-*d*_6_.


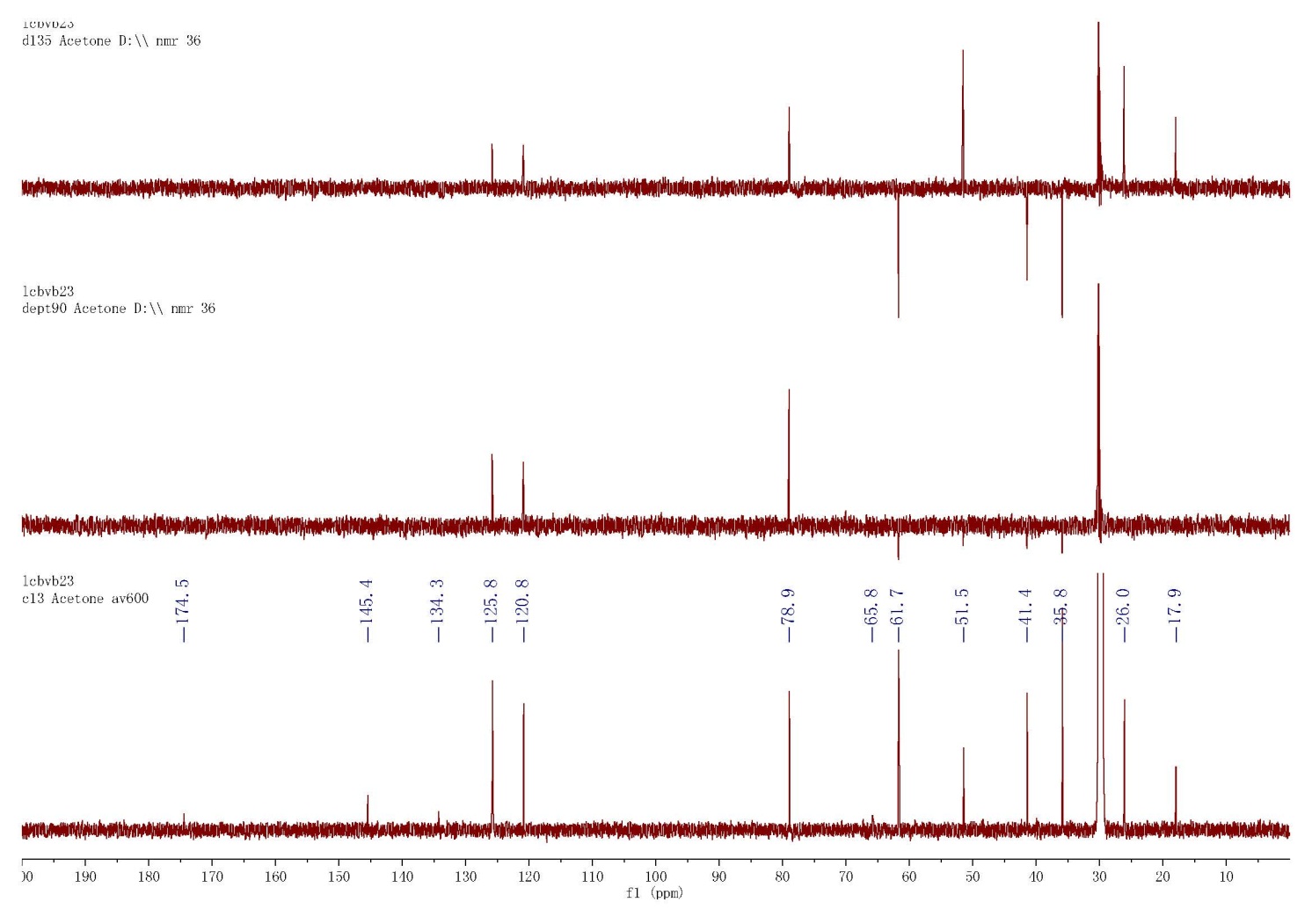


**Figure 3S.**HSQC (600 MHz) spectrum of compound **1** in acetone-*d*_6_.


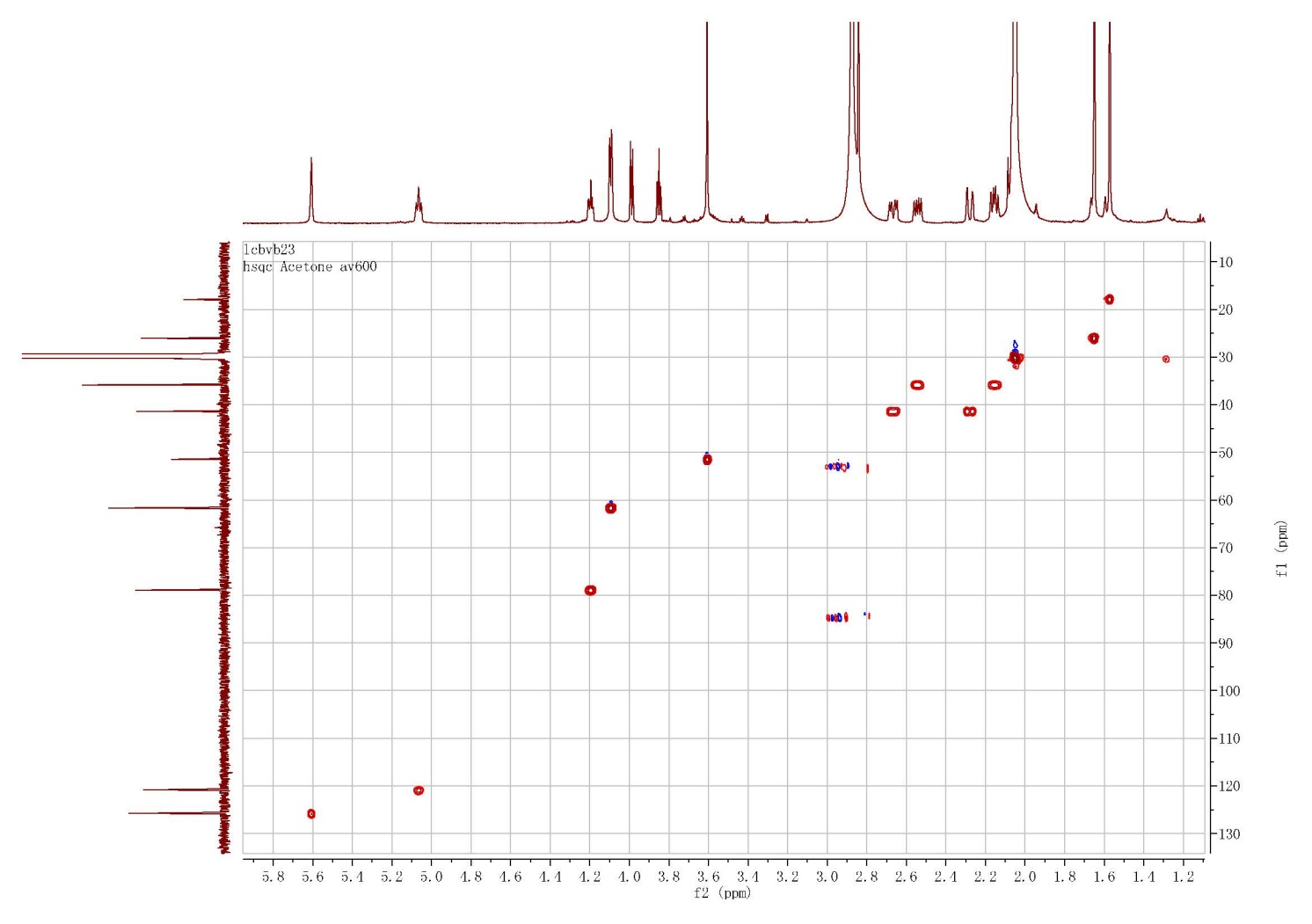


**Figure 4S.**HMBC (600 MHz) spectrum of compound **1** in acetone-*d*_6_.


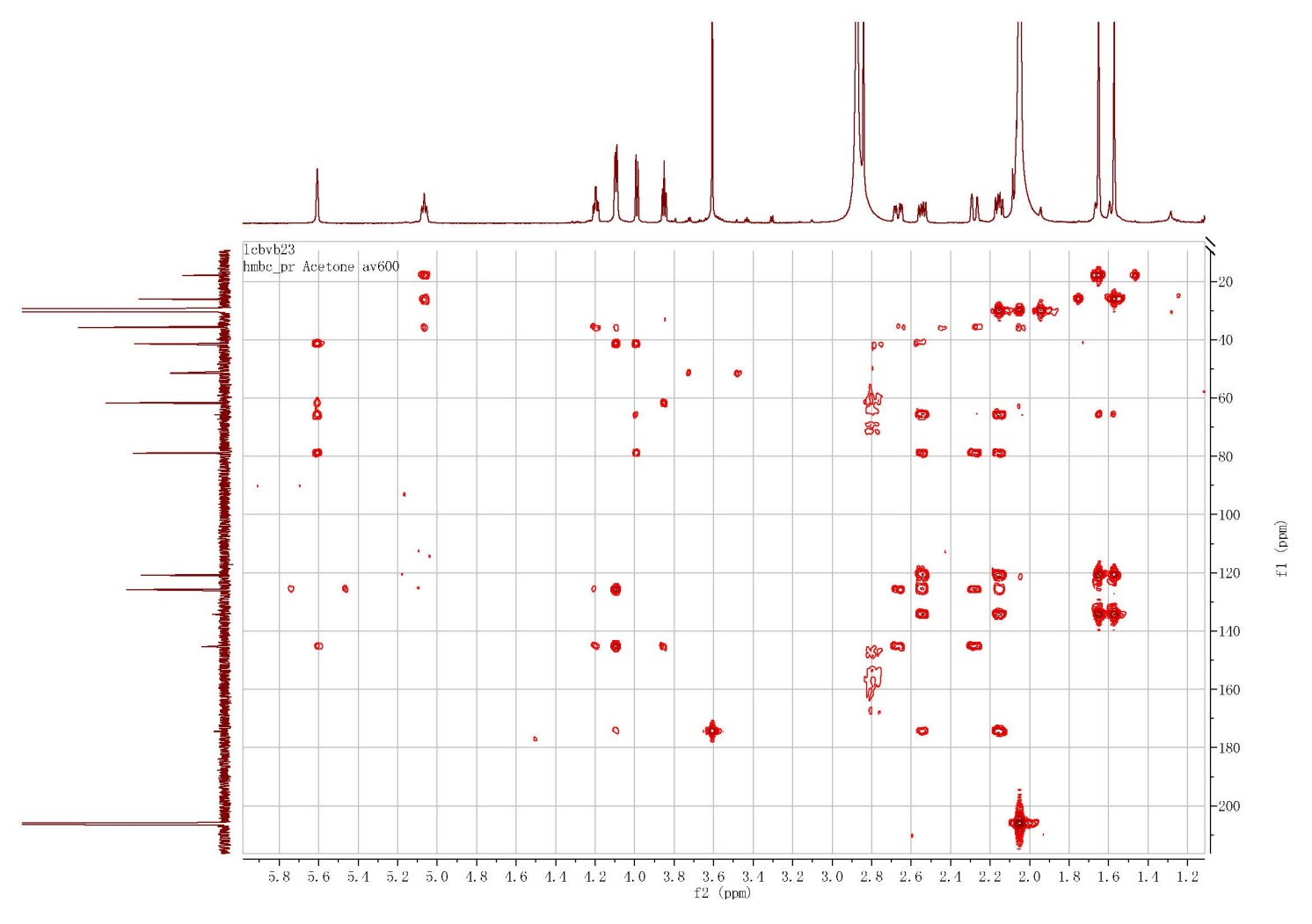


**Figure 5S.**^1^H-^1^H COSY (600 MHz) spectrum of compound **1** in acetone-*d*_6_.


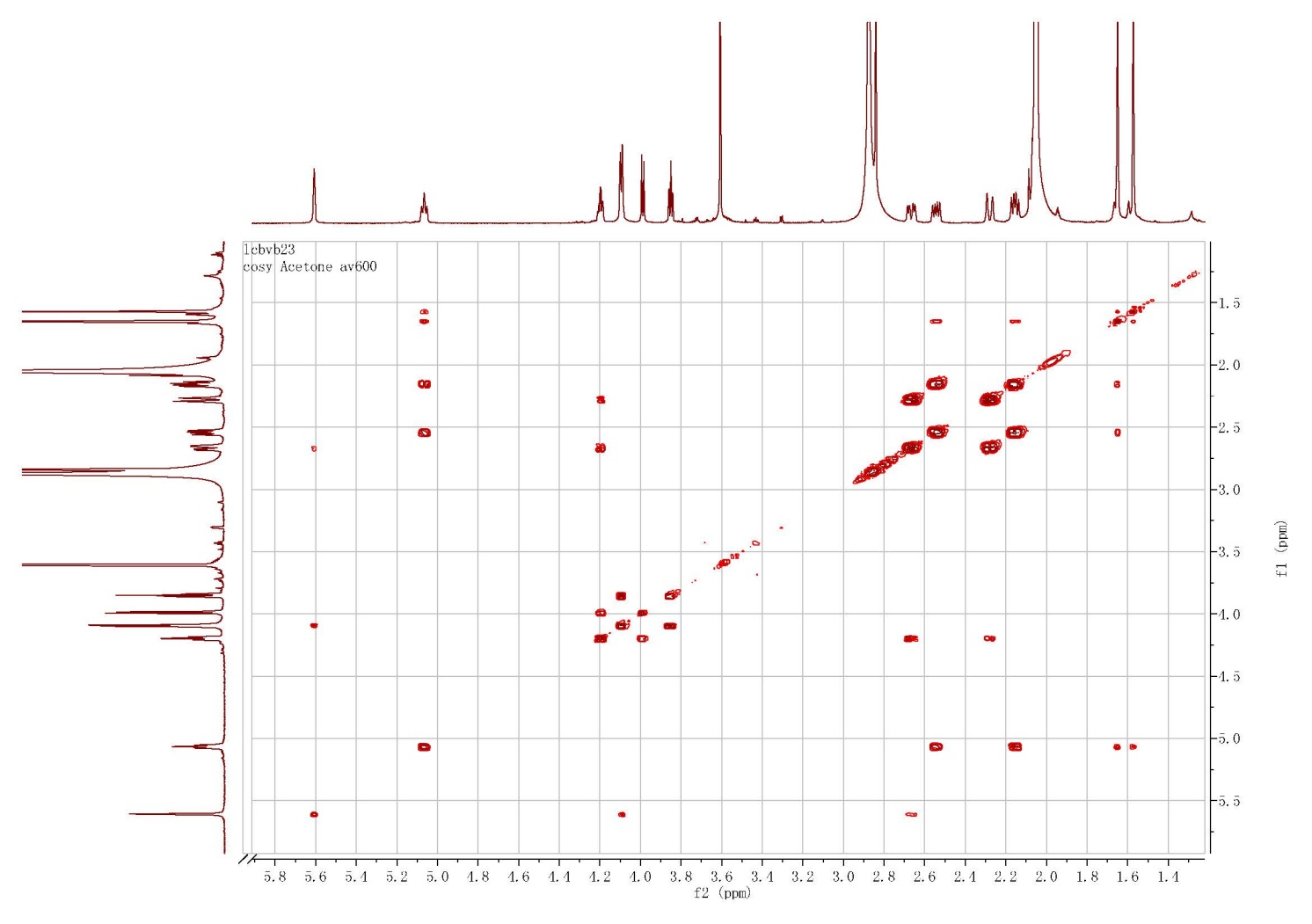


**Figure 6S.**ROESY (600 MHz) spectrum of compound **1** in acetone-*d*_6_.


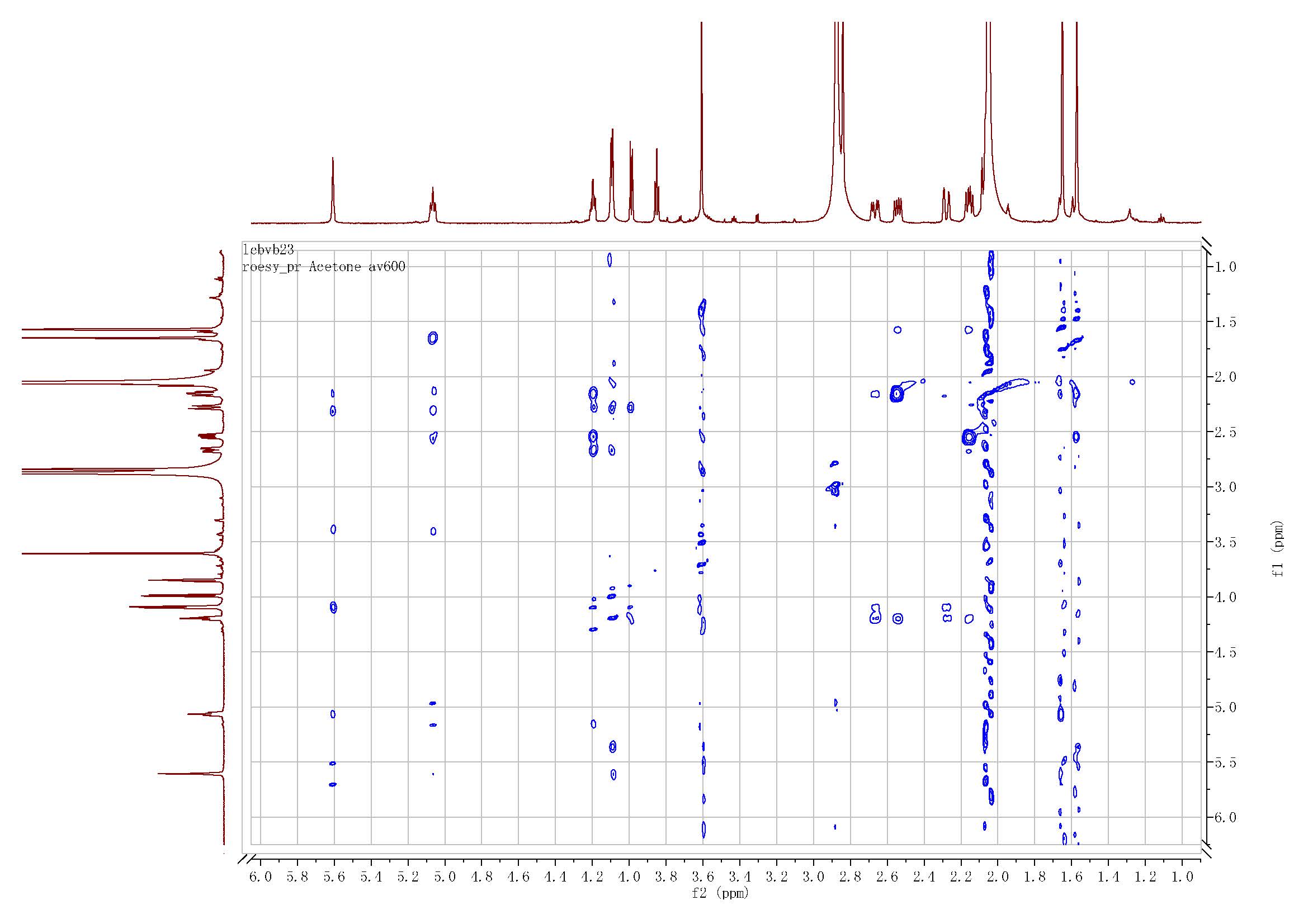


**Figure 7S.**HREIMS spectrum of compound **1**.


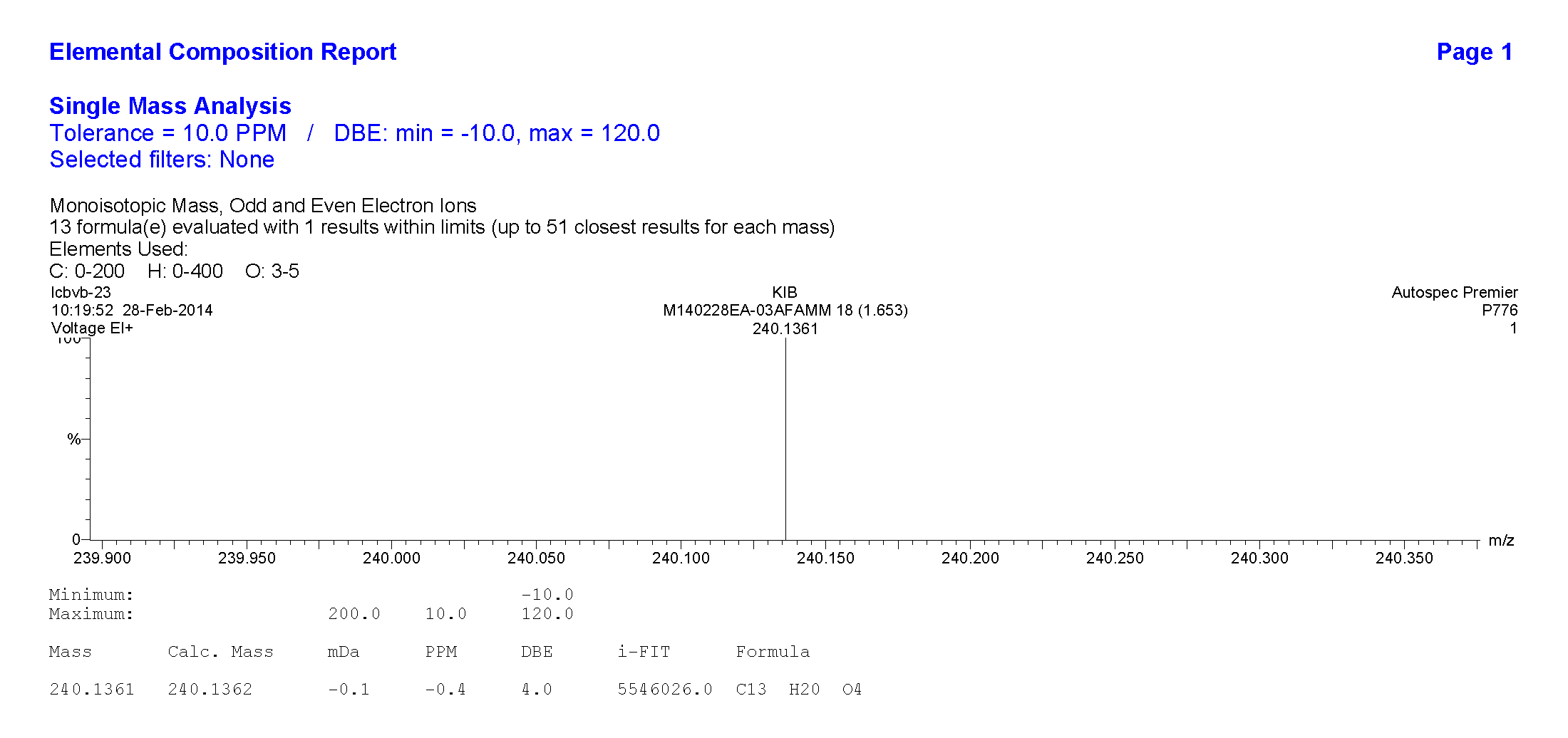


**Figure 8S**.^1^H NMR (600 MHz) spectrum of compound **2** in acetone-*d*_6_.


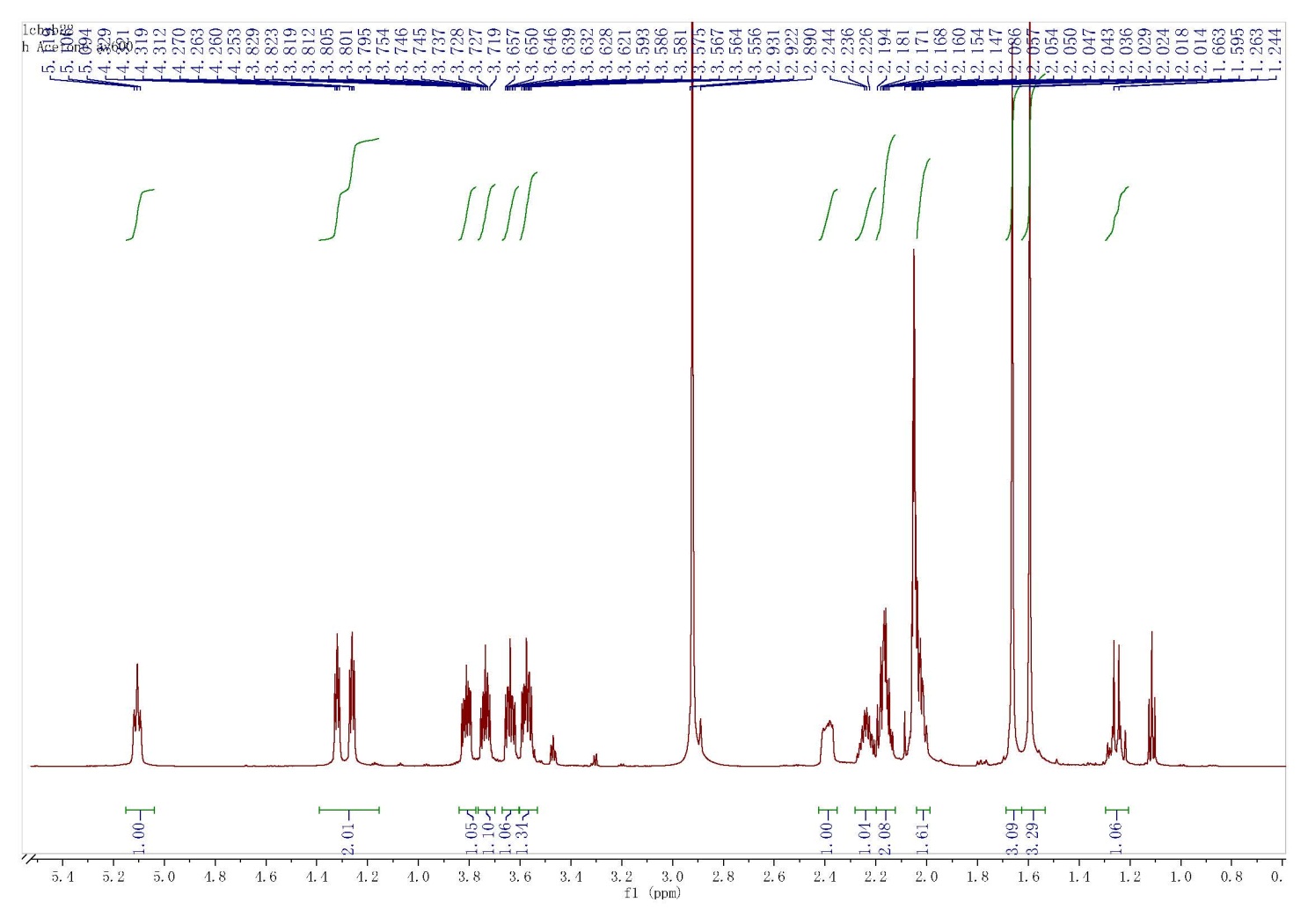


**Figure 9S**.^13^C NMR(150 MHz) and DEPT spectra of compound **2** in acetone-*d*_6_.


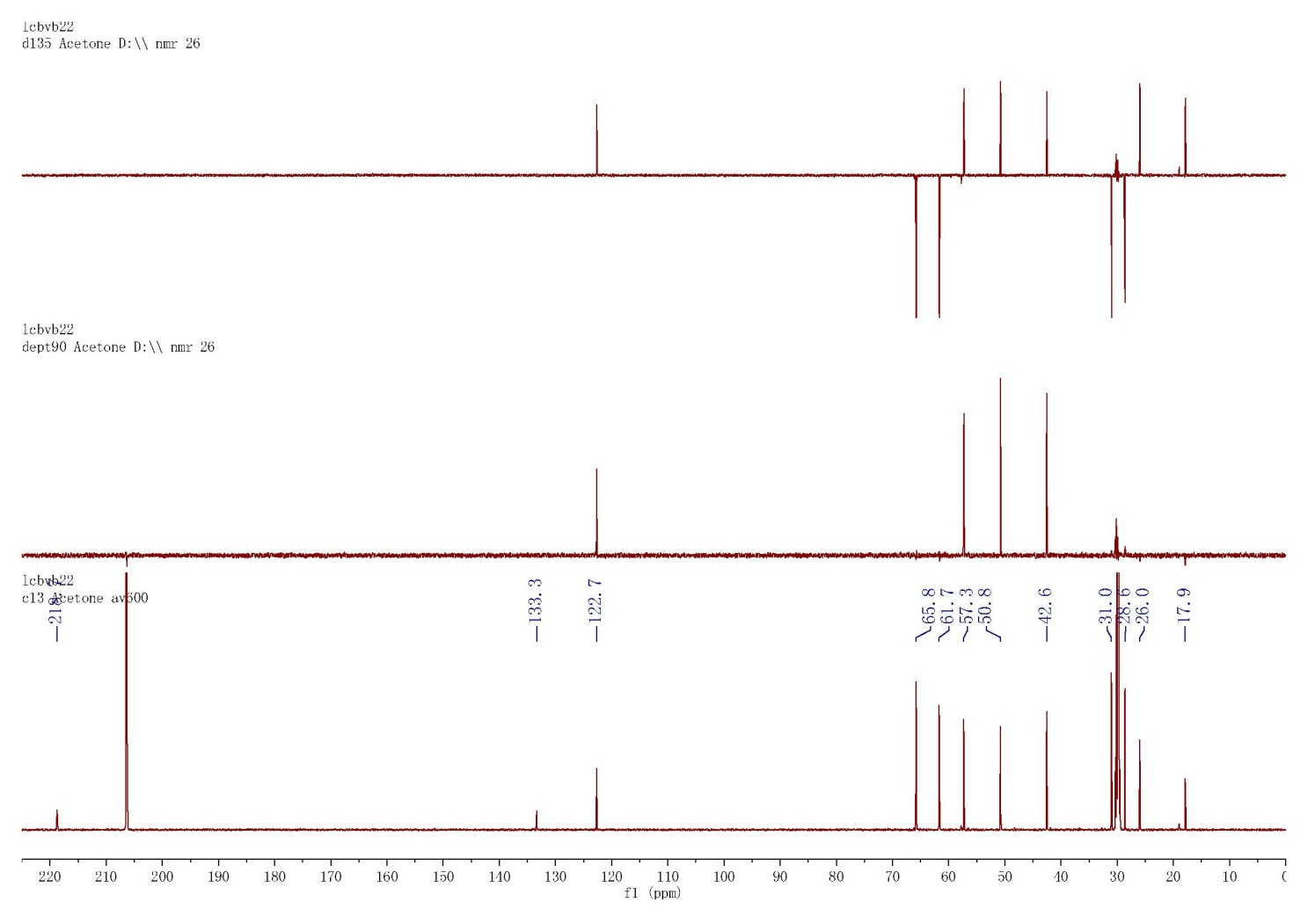


**Figure 10S.**HSQC (600 MHz) spectrum of compound **2** in acetone-*d*_6_.


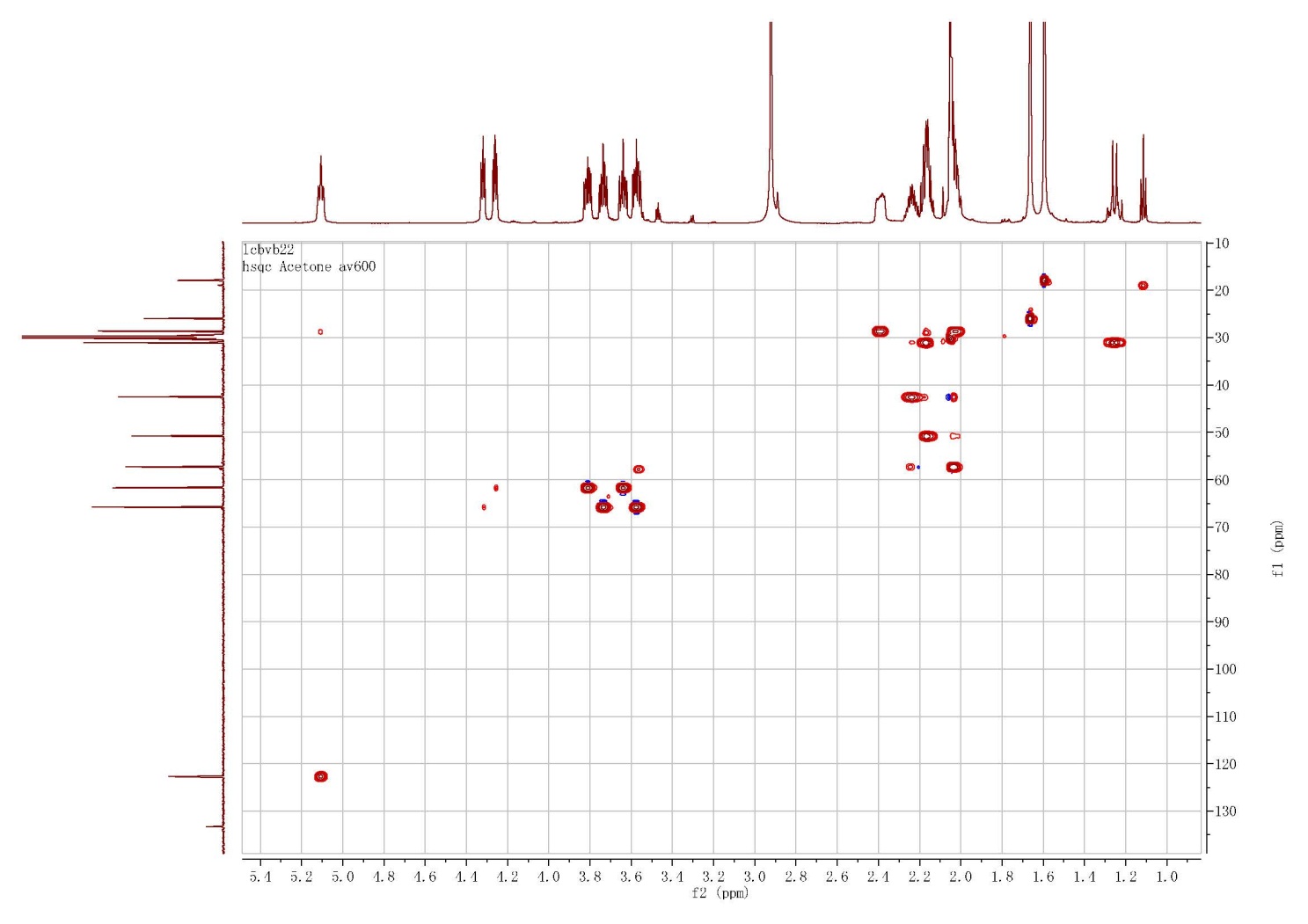


**Figure 11S.**HMBC (600 MHz) spectrum of compound **2** in acetone-*d*_6_.


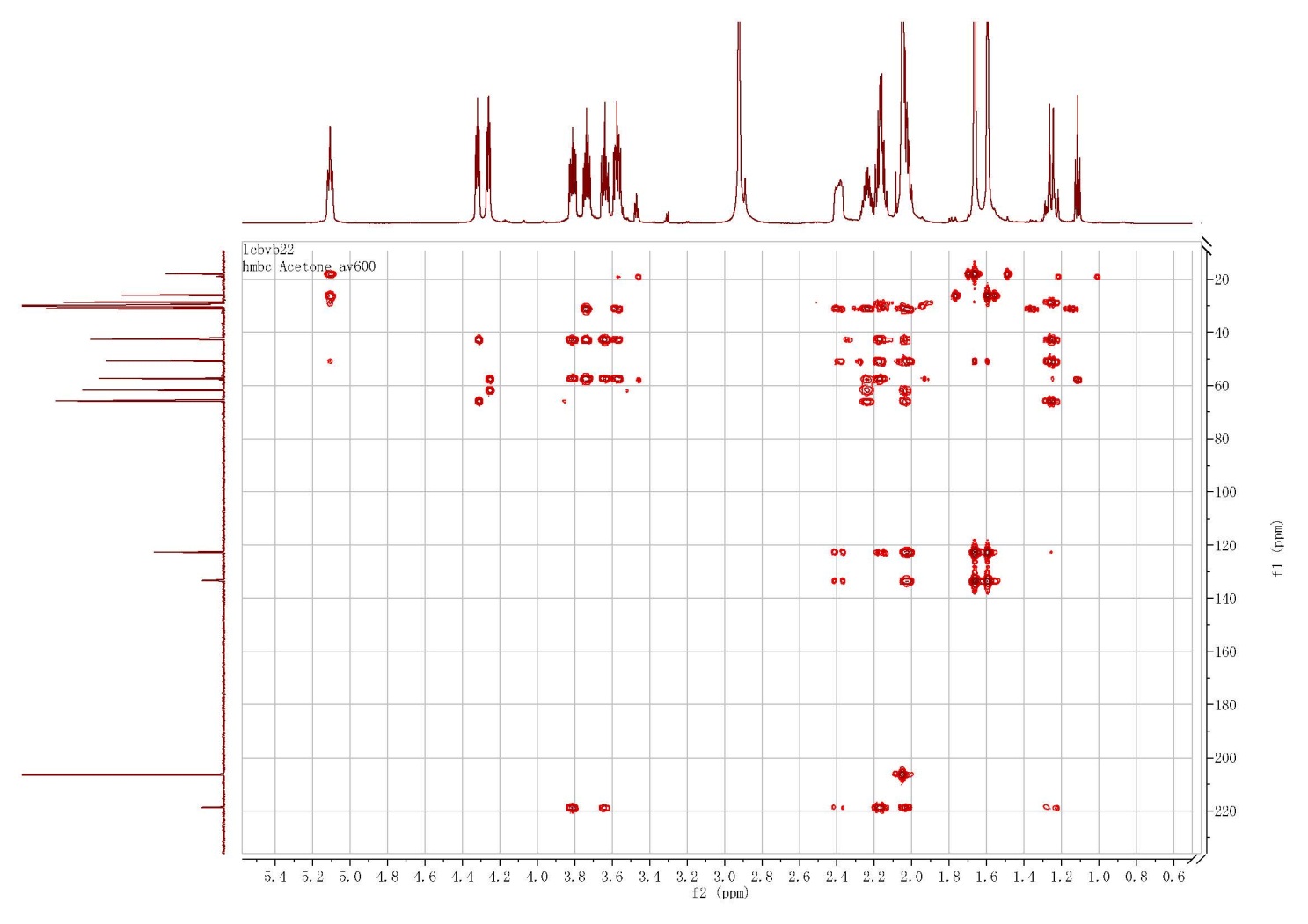


**Figure 12S.**^1^H-^1^H COSY (600 MHz) spectrum of compound **2** in acetone-*d*_6_.


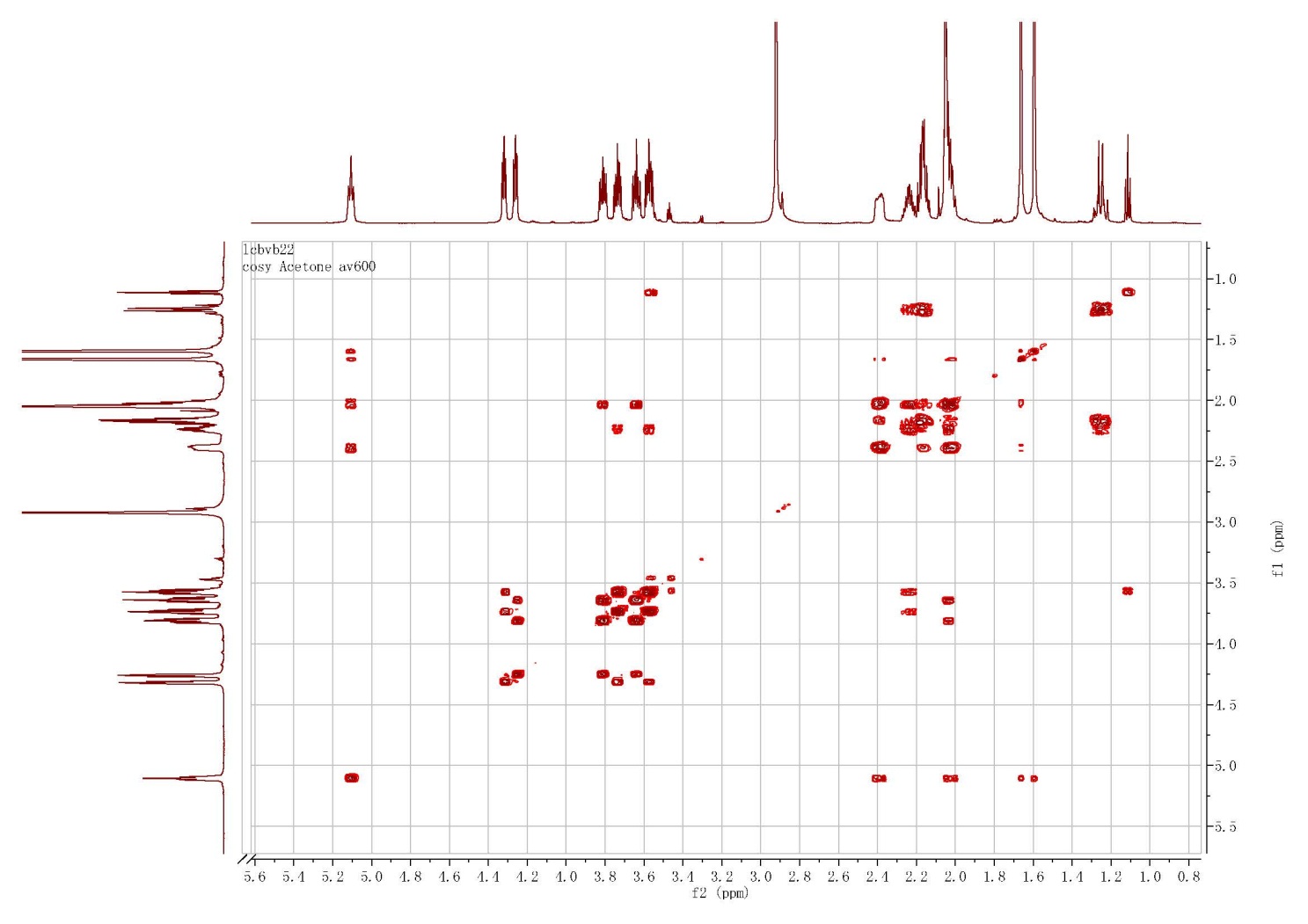


**Figure 13S.**ROESY (600 MHz) spectrum of compound **2** in acetone-*d*_6_.


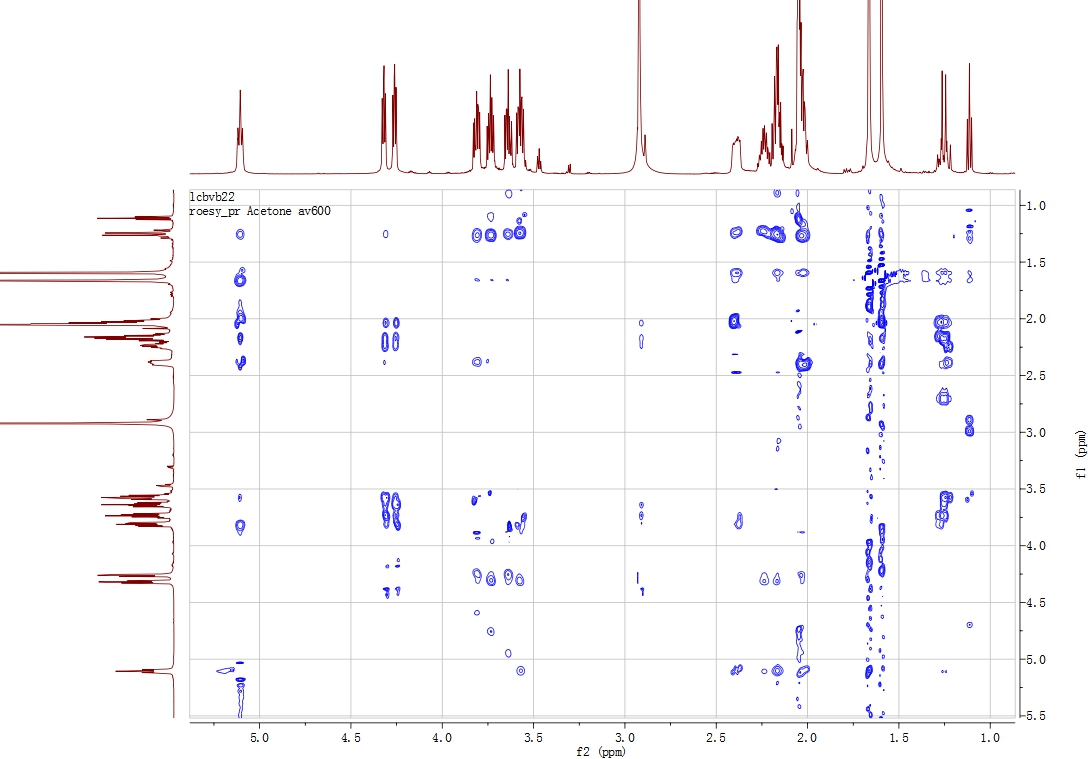


**Figure 14S.**HREIMS spectrum of compound **2**.

**
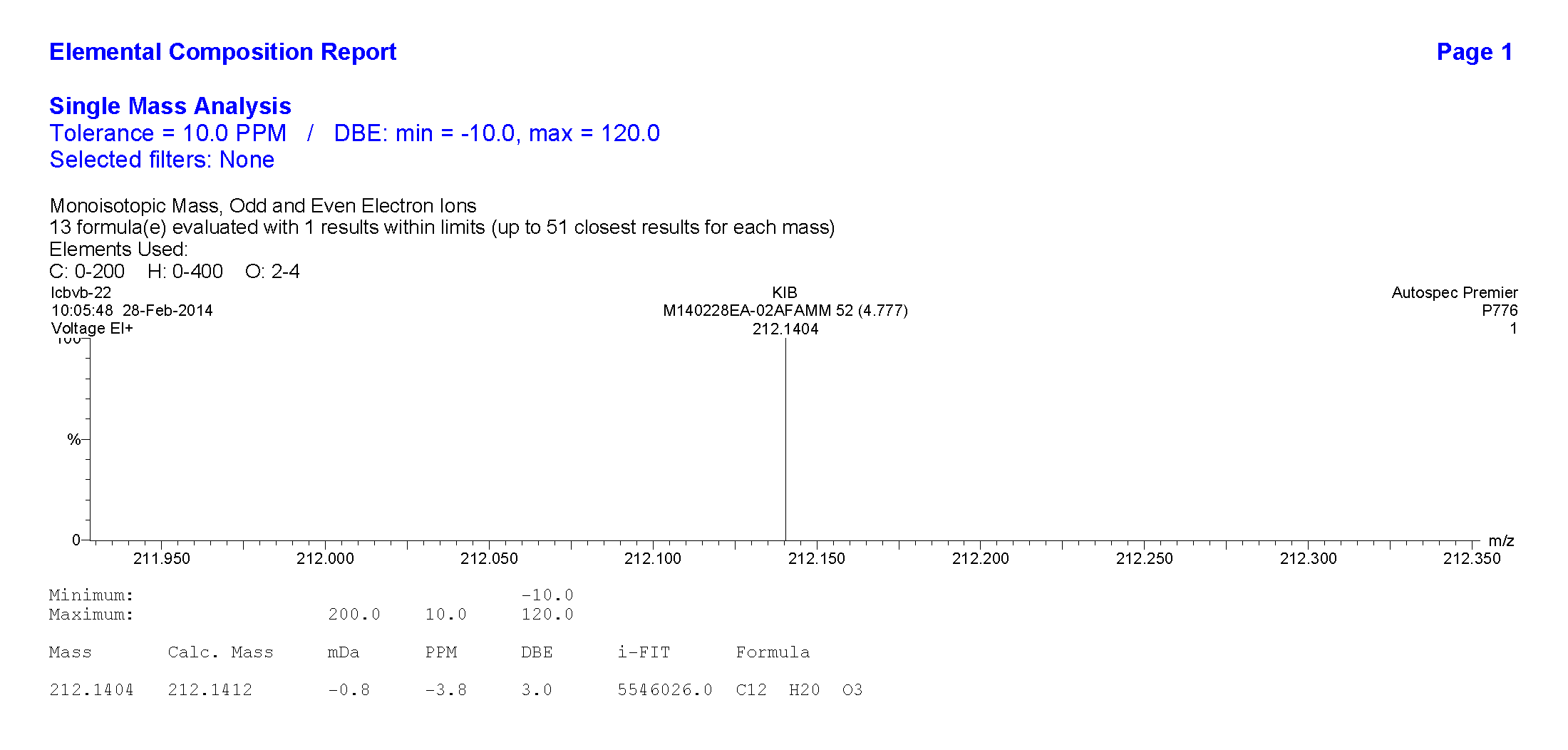
**

**Figure 15S**.^1^H NMR (600 MHz) spectrum of compound **3** in CDCl_3_.


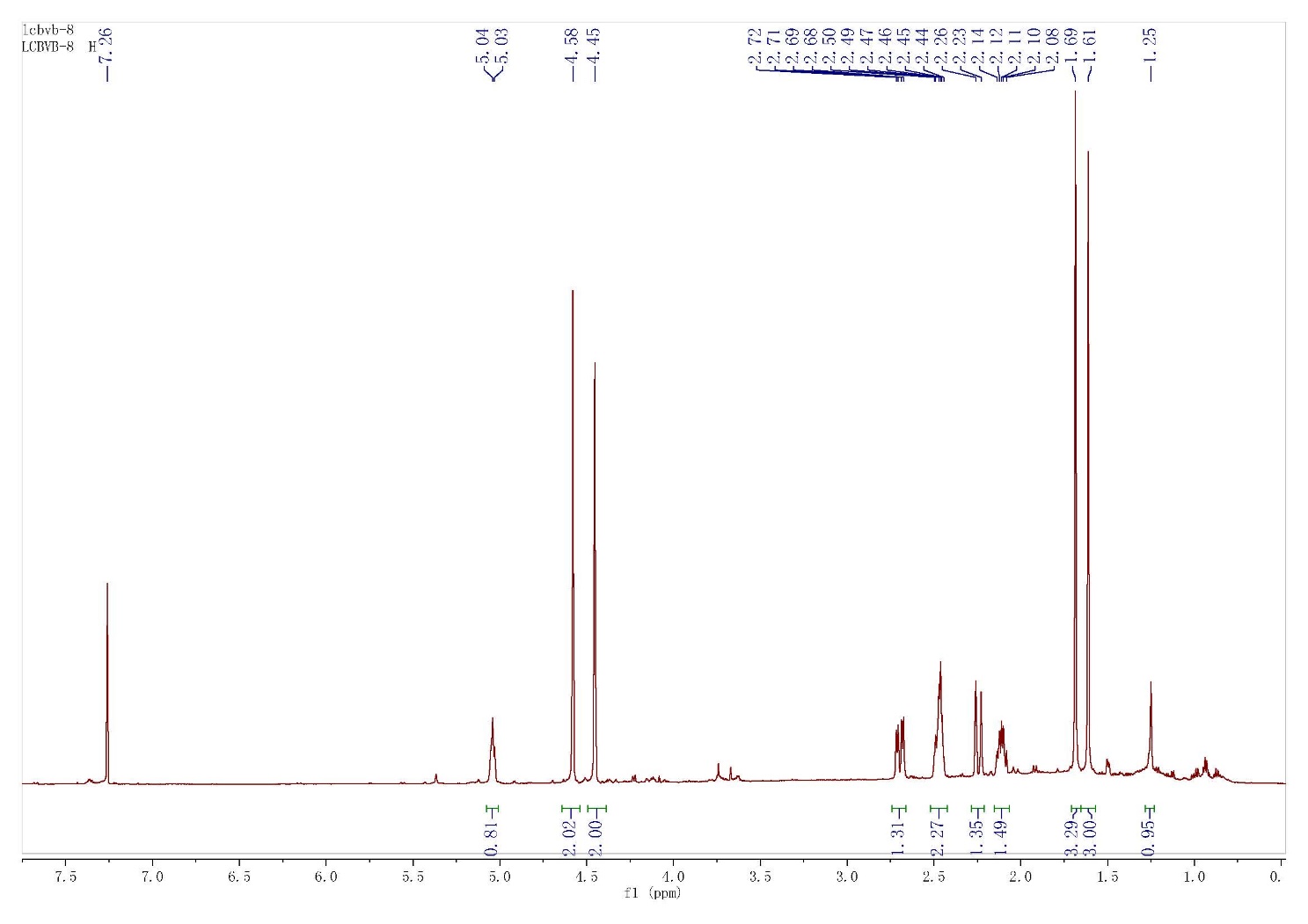


**Figure 16S**.^13^C NMR(150 MHz) and DEPT spectra of compound **3** in CDCl_3_.


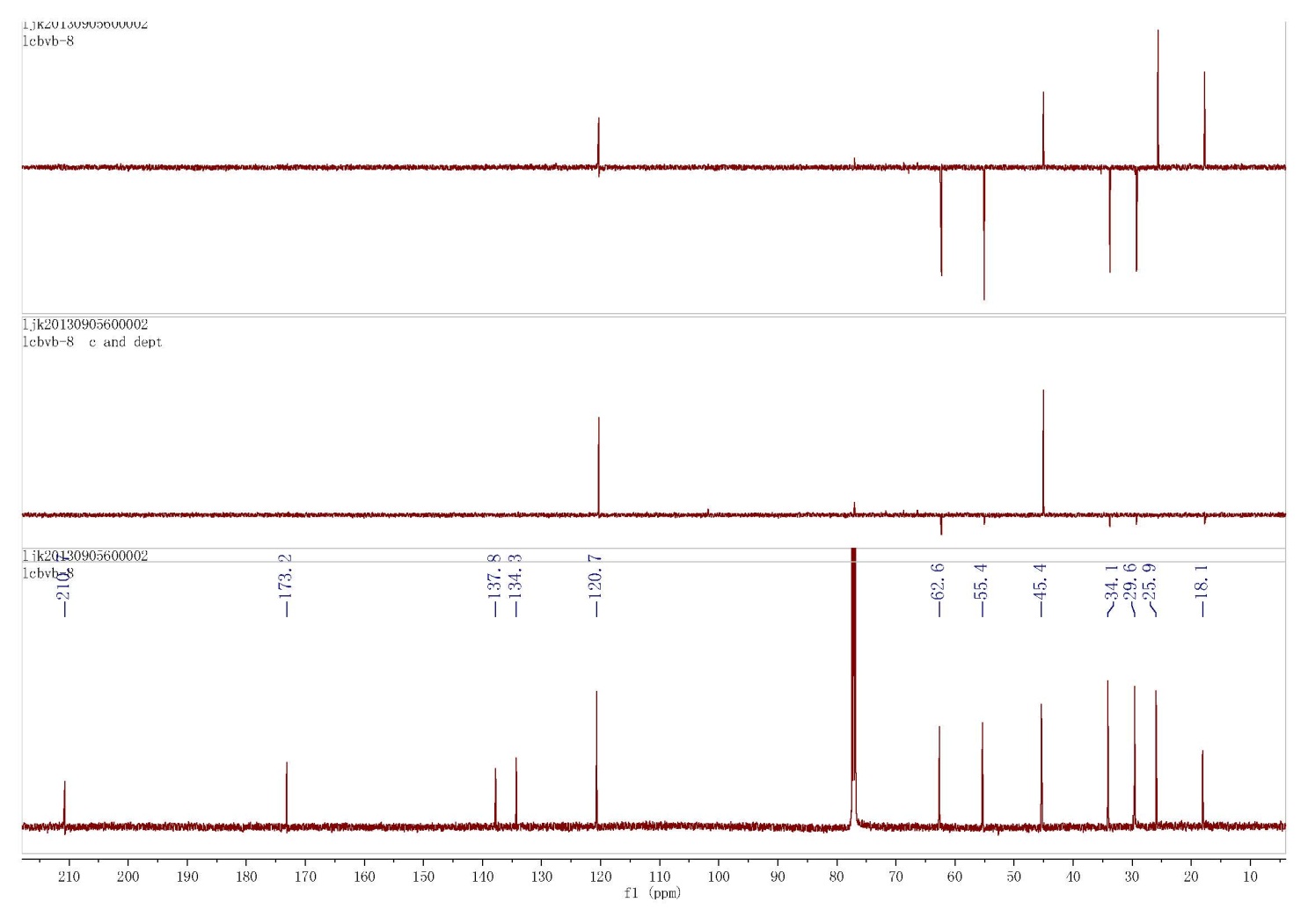


**Figure 17S.**HSQC (600 MHz) spectrum of compound **3** in CDCl_3_.


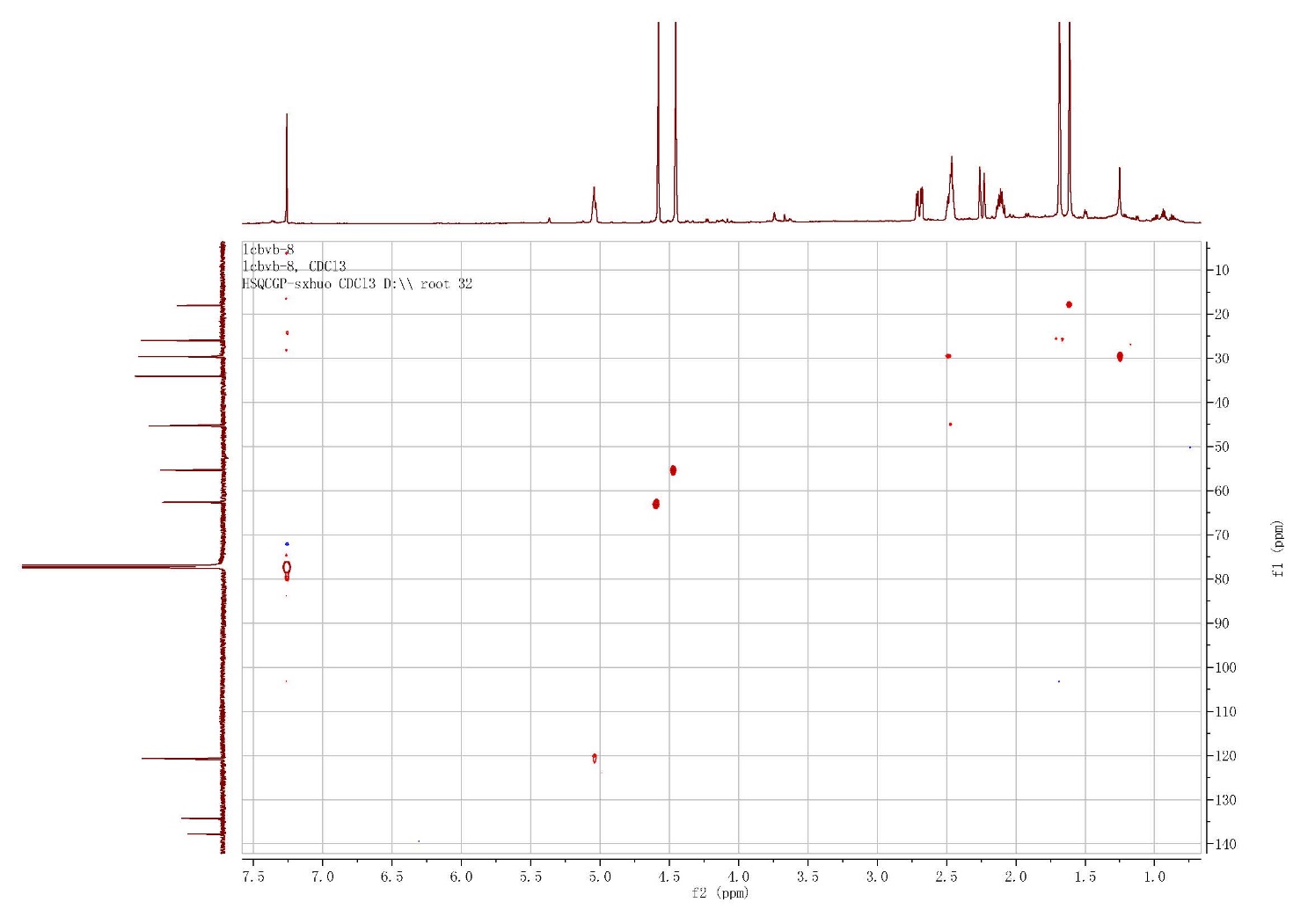


**Figure 18S.**HMBC (600 MHz) spectrum of compound **3** in CDCl_3_.


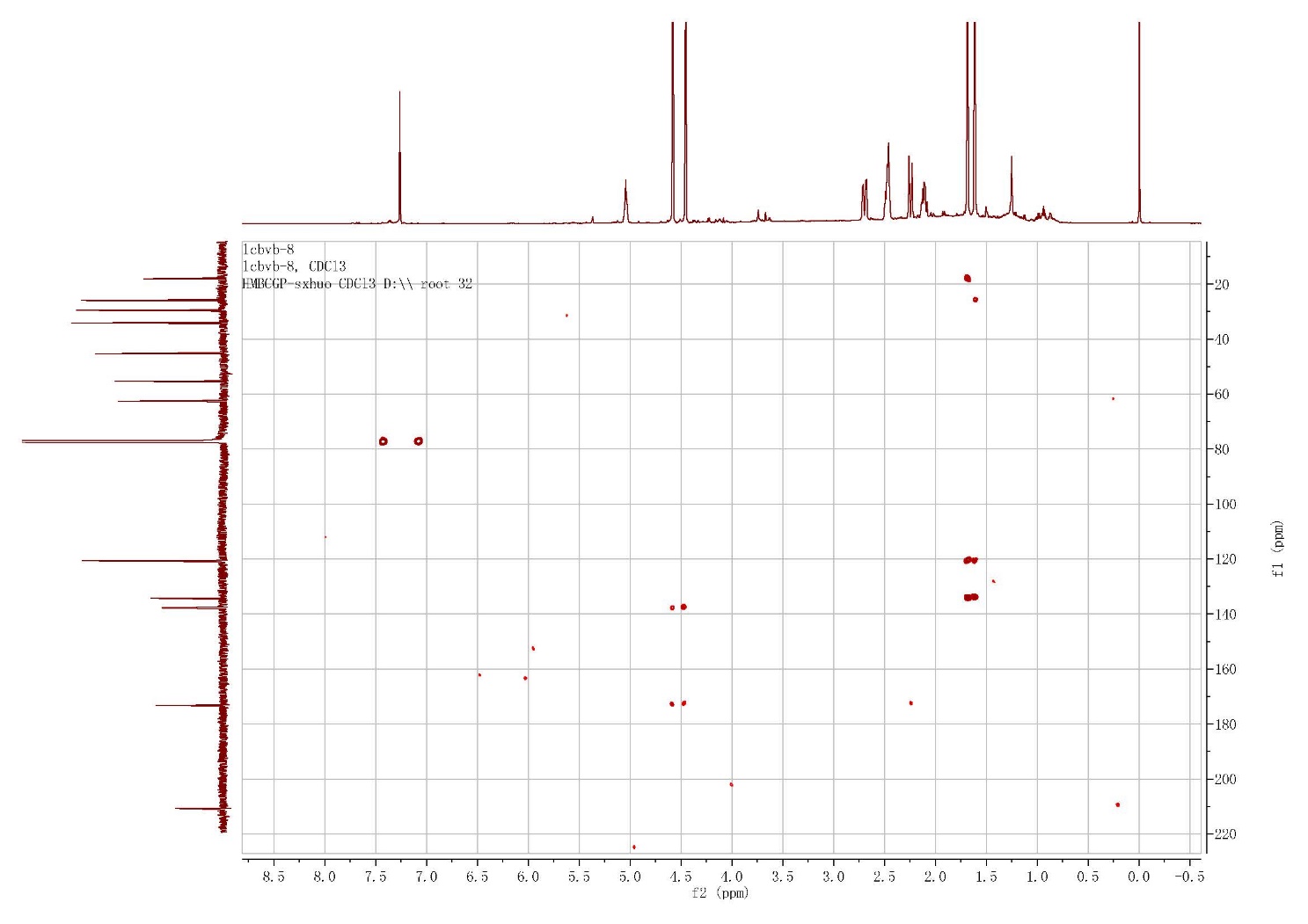


**Figure 19S.**^1^H-^1^H COSY (600 MHz) spectrum of compound **3** in CDCl_3_.


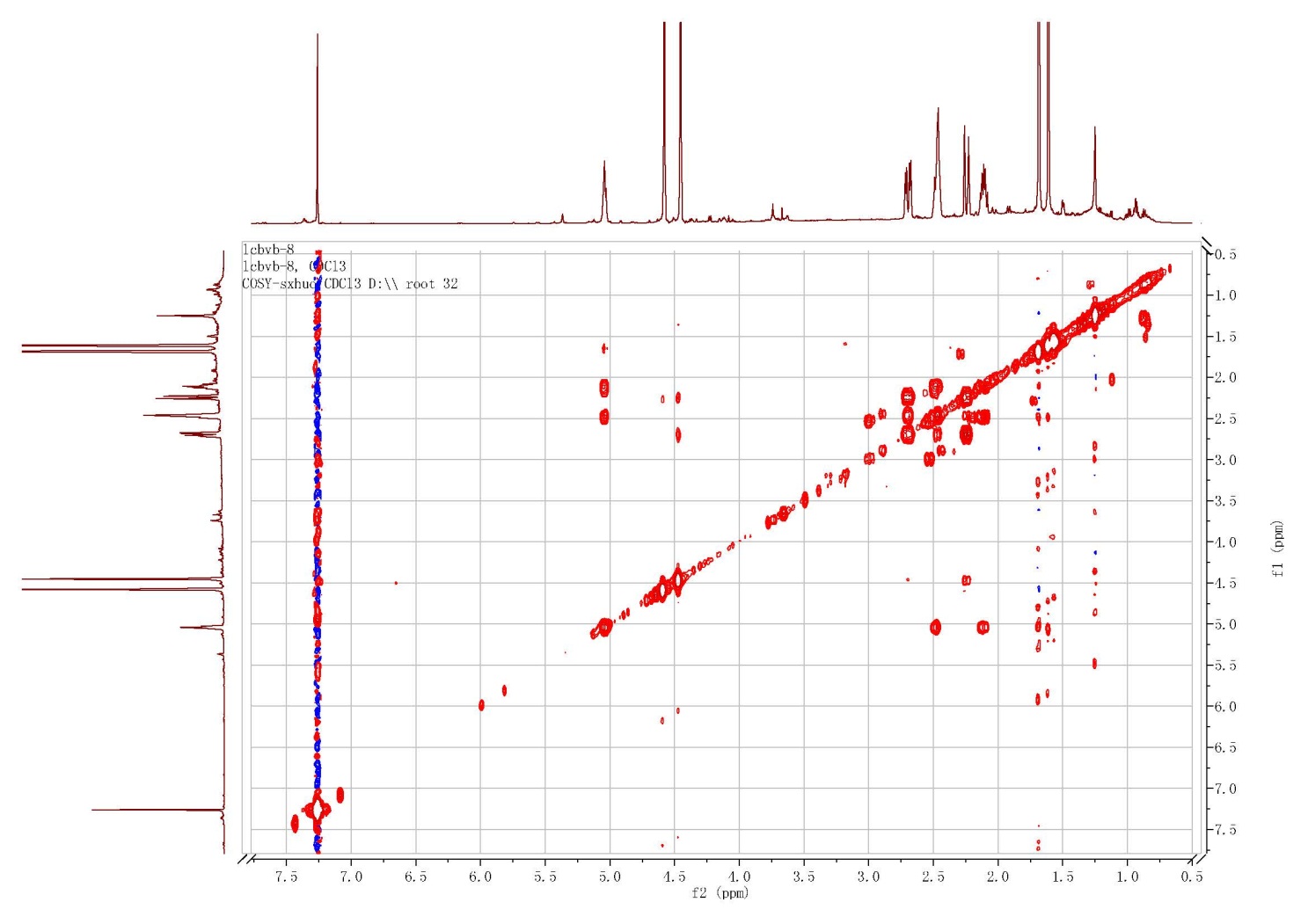


**Figure 20S.**ROESY (600 MHz) spectrum of compound **3** in CDCl_3_.


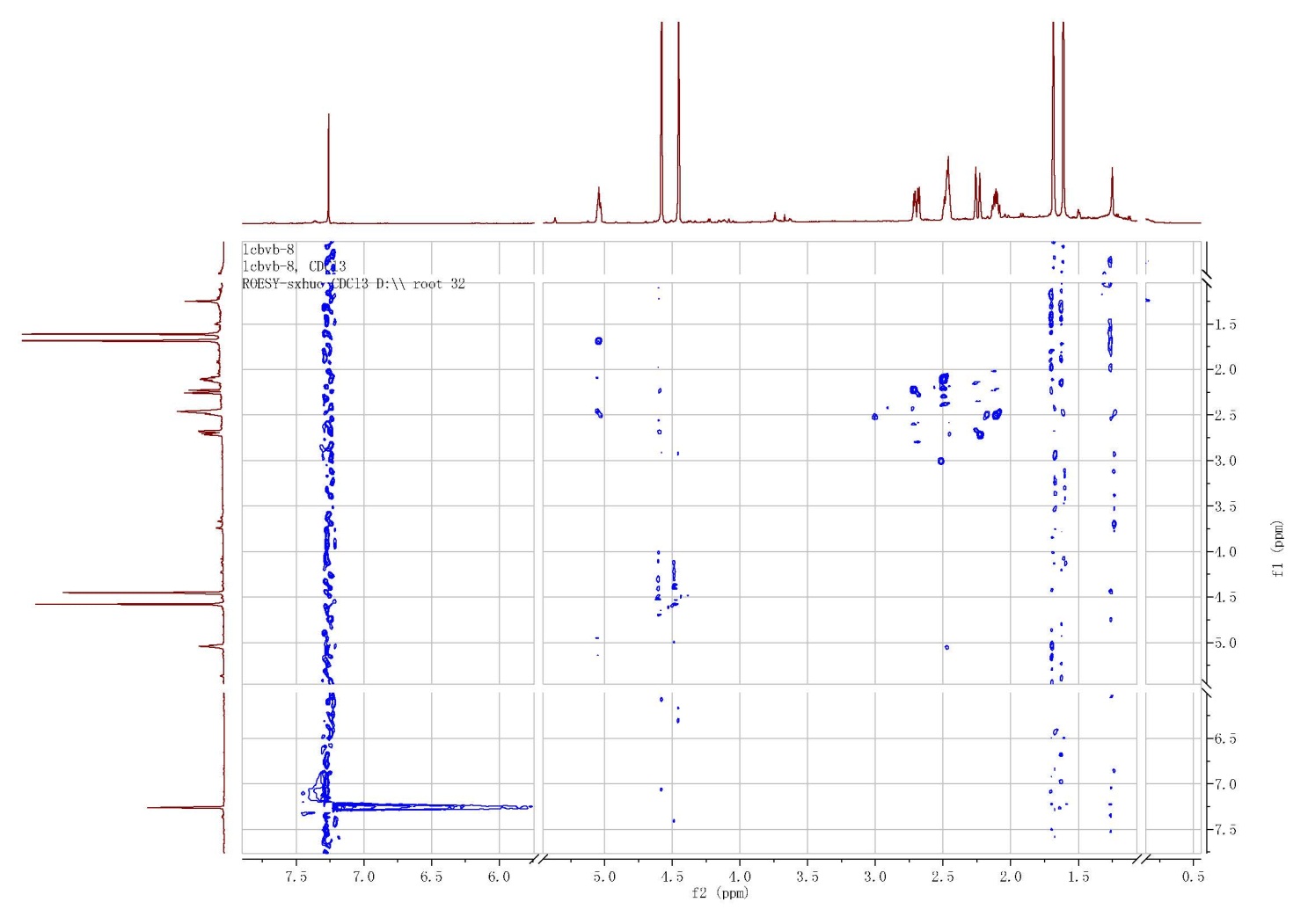


**Figure 21S.**HREIMS spectrum of compound **3**.


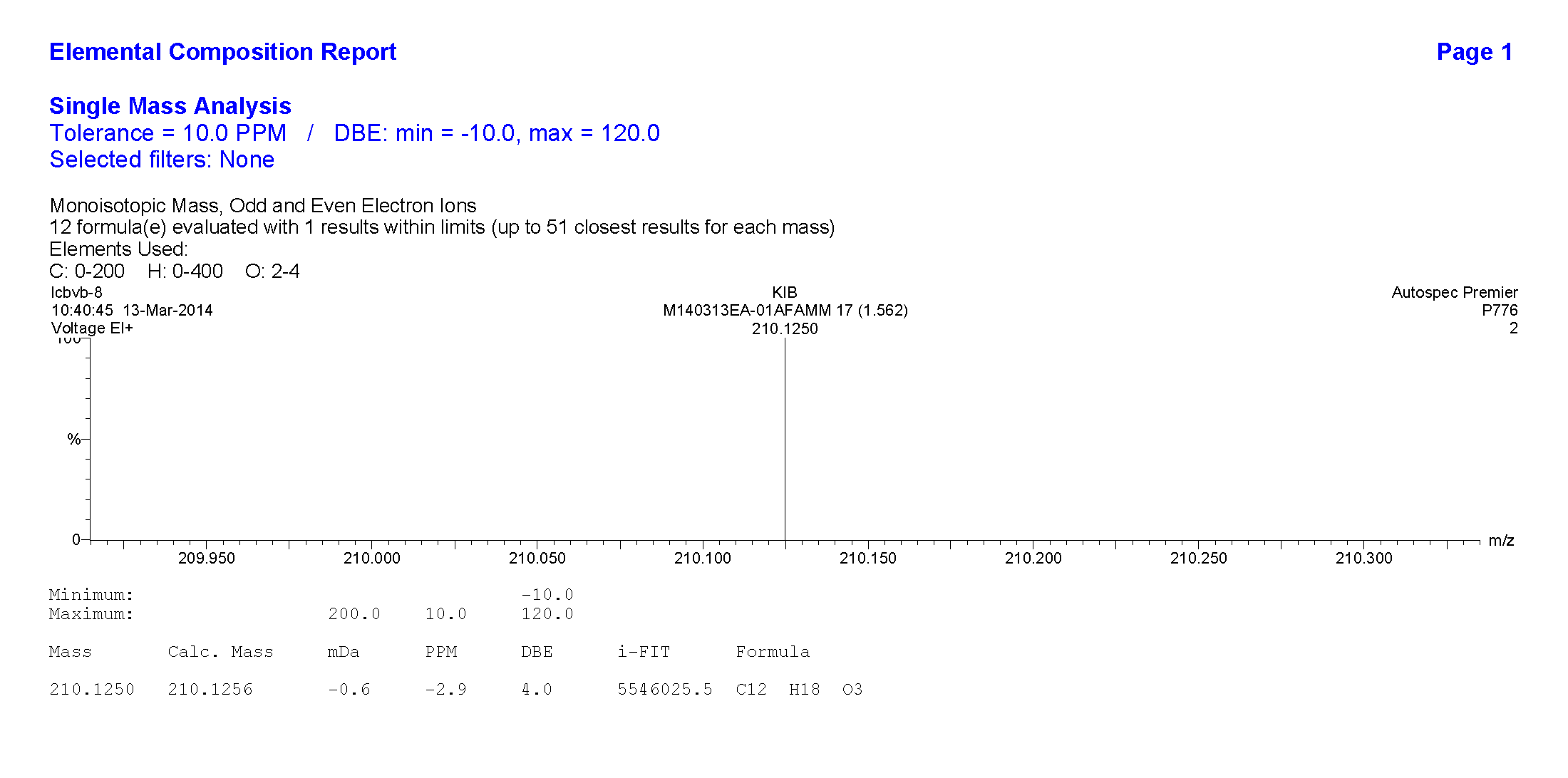


**Figure 22S**.^1^H NMR (600 MHz) spectrum of compound **4** in acetone-*d*_6_.


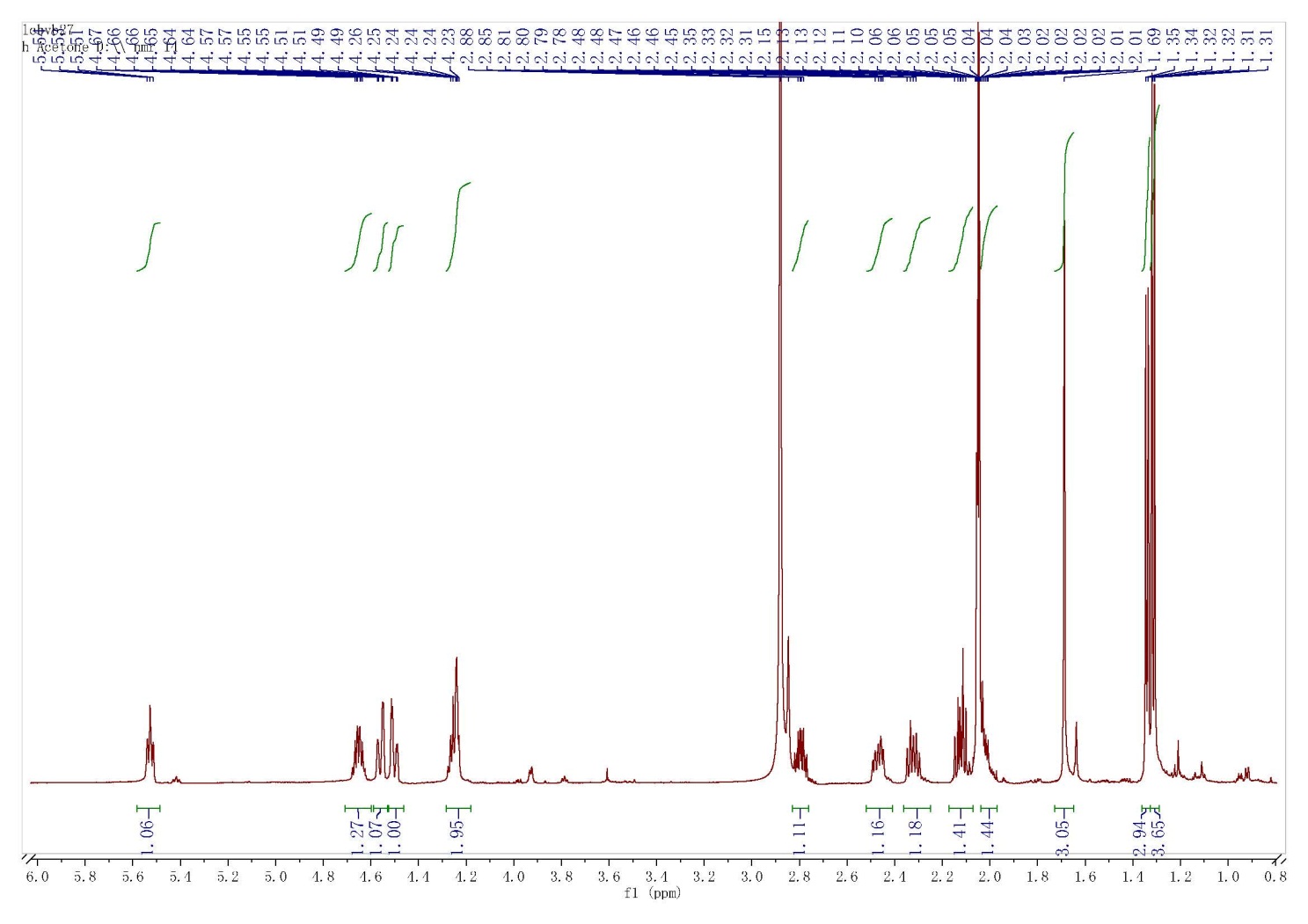


**Figure 23S**.^13^C NMR(150 MHz) and DEPT spectra of compound **4** in acetone-*d*_6_.


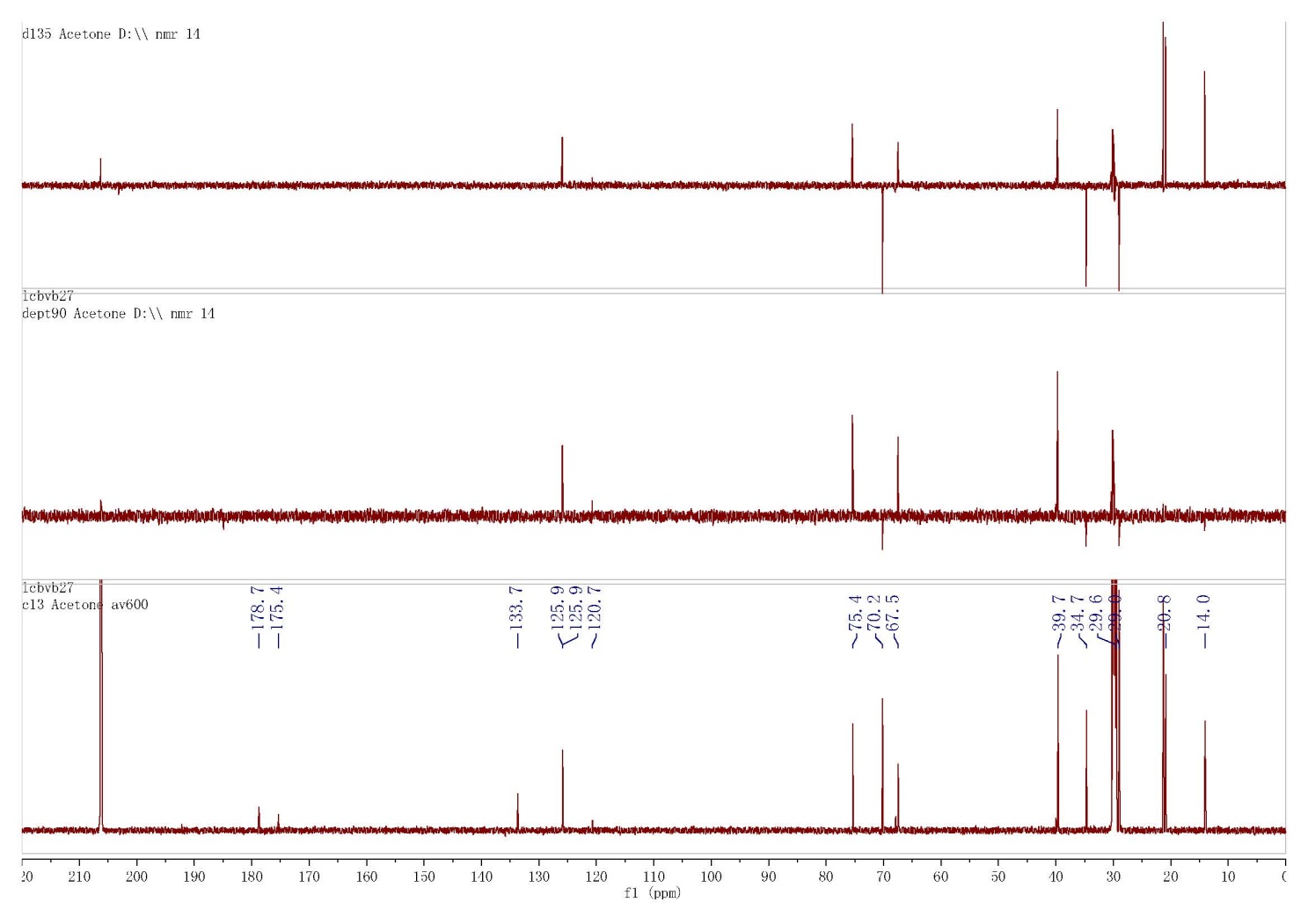


**Figure 24S.**HSQC (600 MHz) spectrum of compound **4** in acetone-*d*_6_.


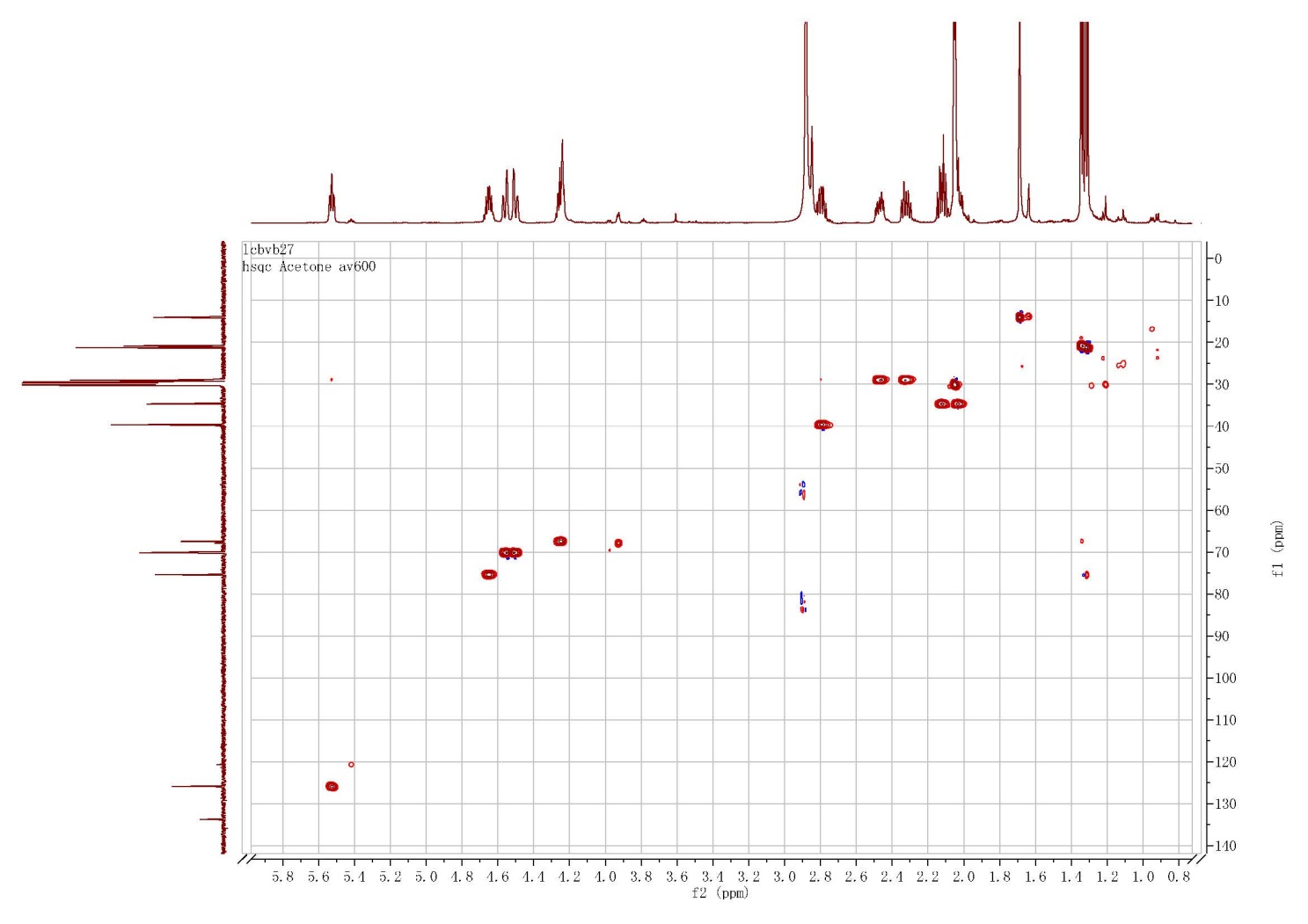


**Figure 25S.**HMBC (600 MHz) spectrum of compound **4** in acetone-*d*_6_.


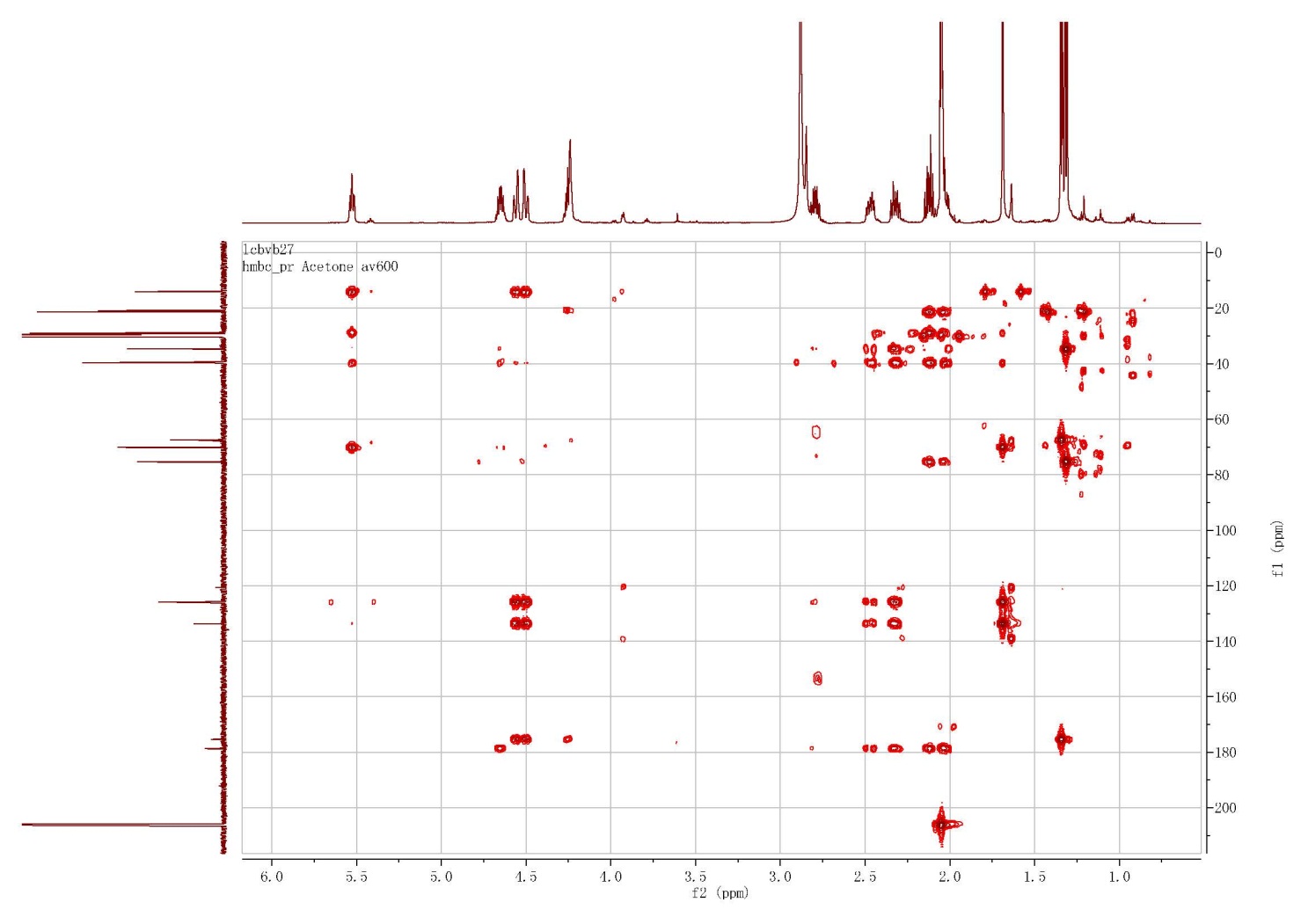


**Figure 26S.**^1^H-^1^H COSY (600 MHz) spectrum of compound **4** in acetone-*d*_6_.


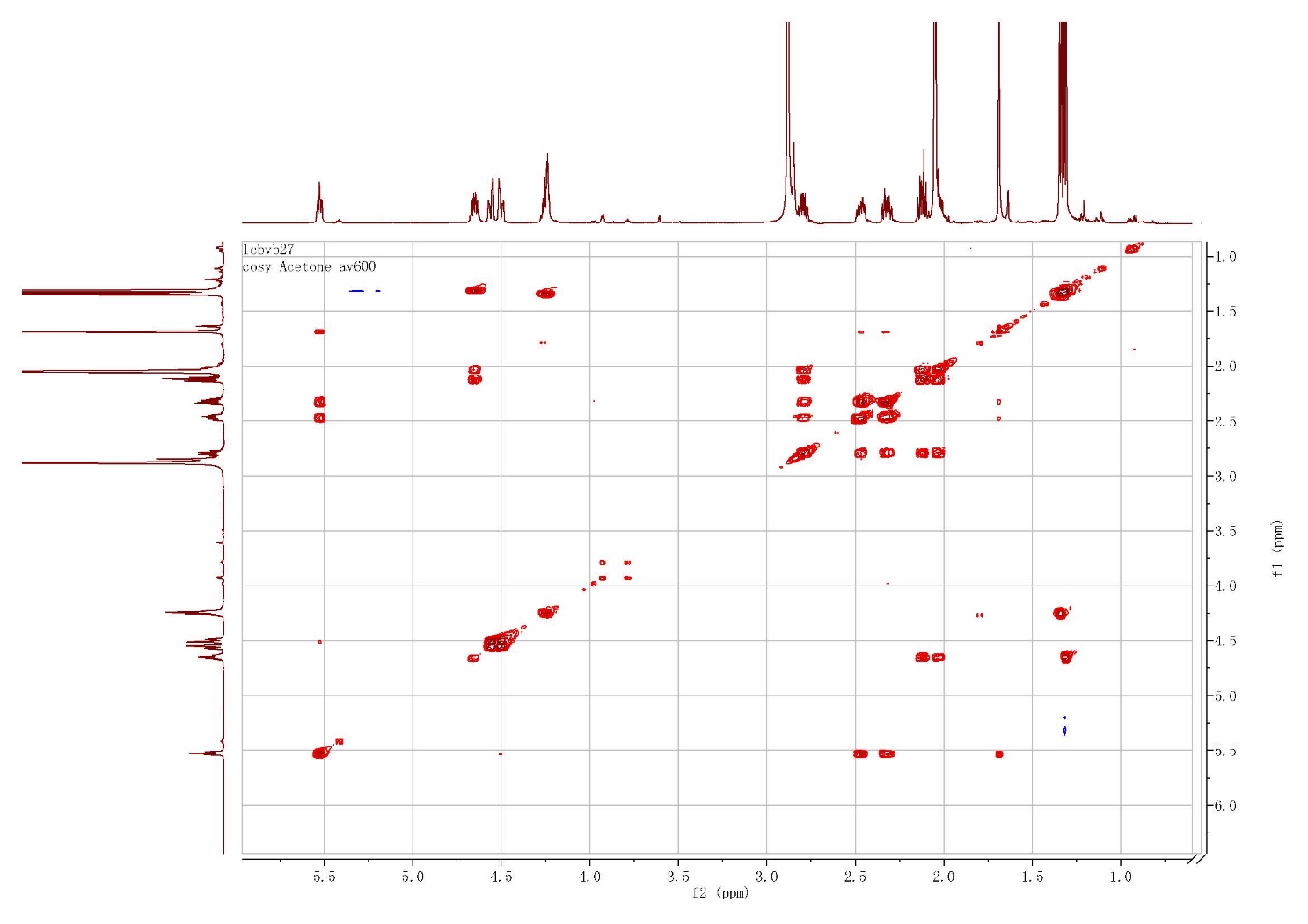


**Figure 27S.**ROESY (600 MHz) spectrum of compound **4** in acetone-*d*_6_.

**
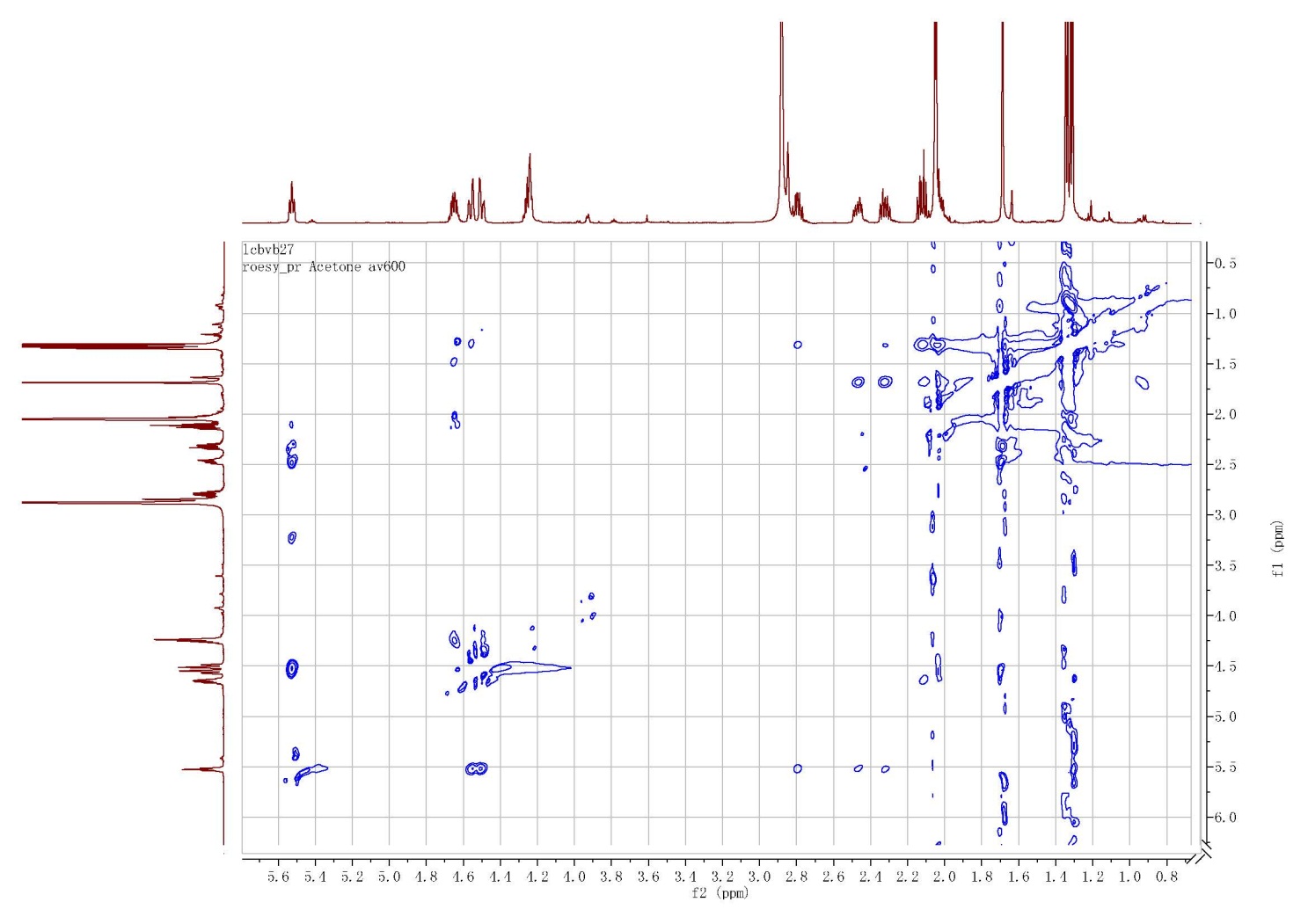
**

**Figure 28S.**HRESIMS spectrum of compound **4**.


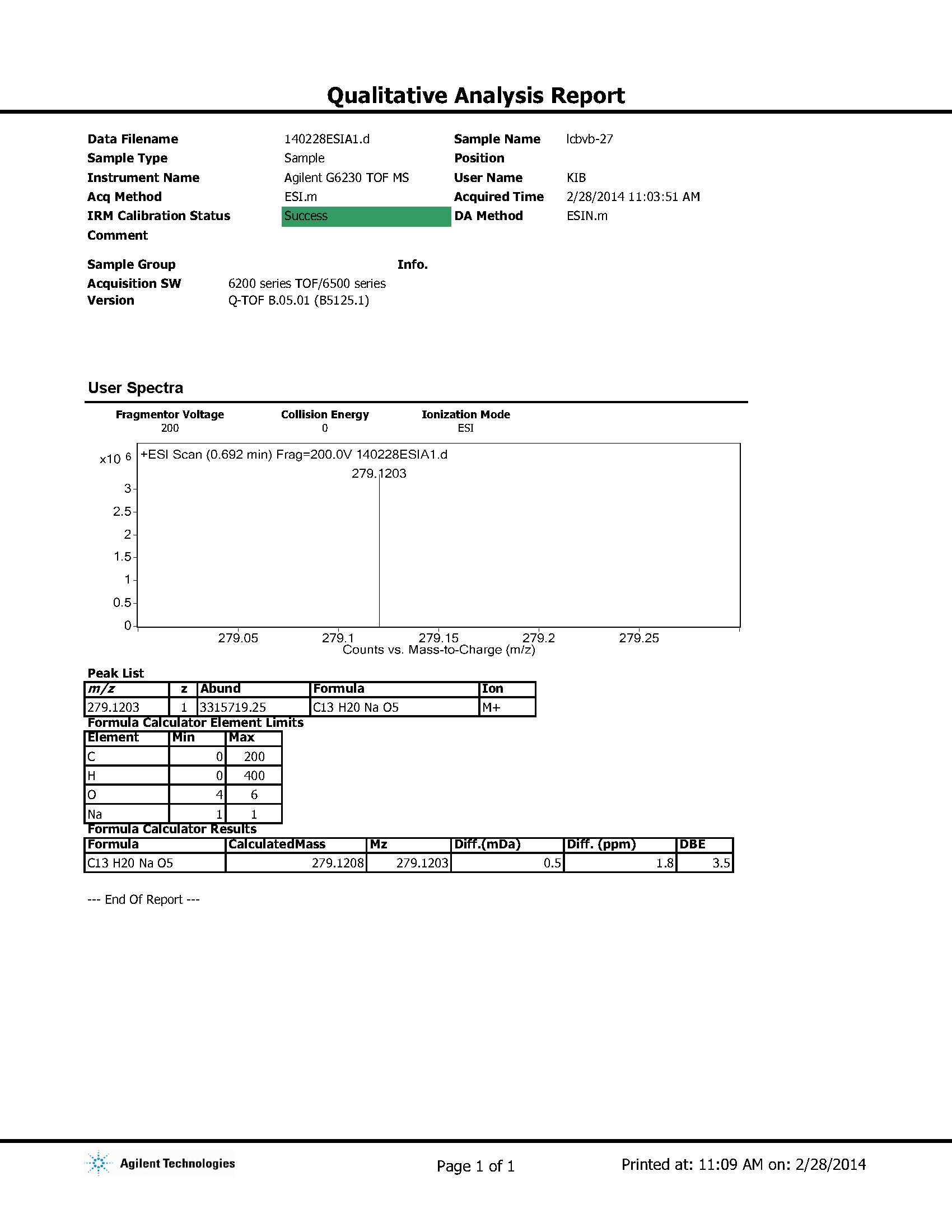


**Figure 29S**.^1^H NMR (800 MHz) spectrum of compound **5** in acetone-*d*_6_.


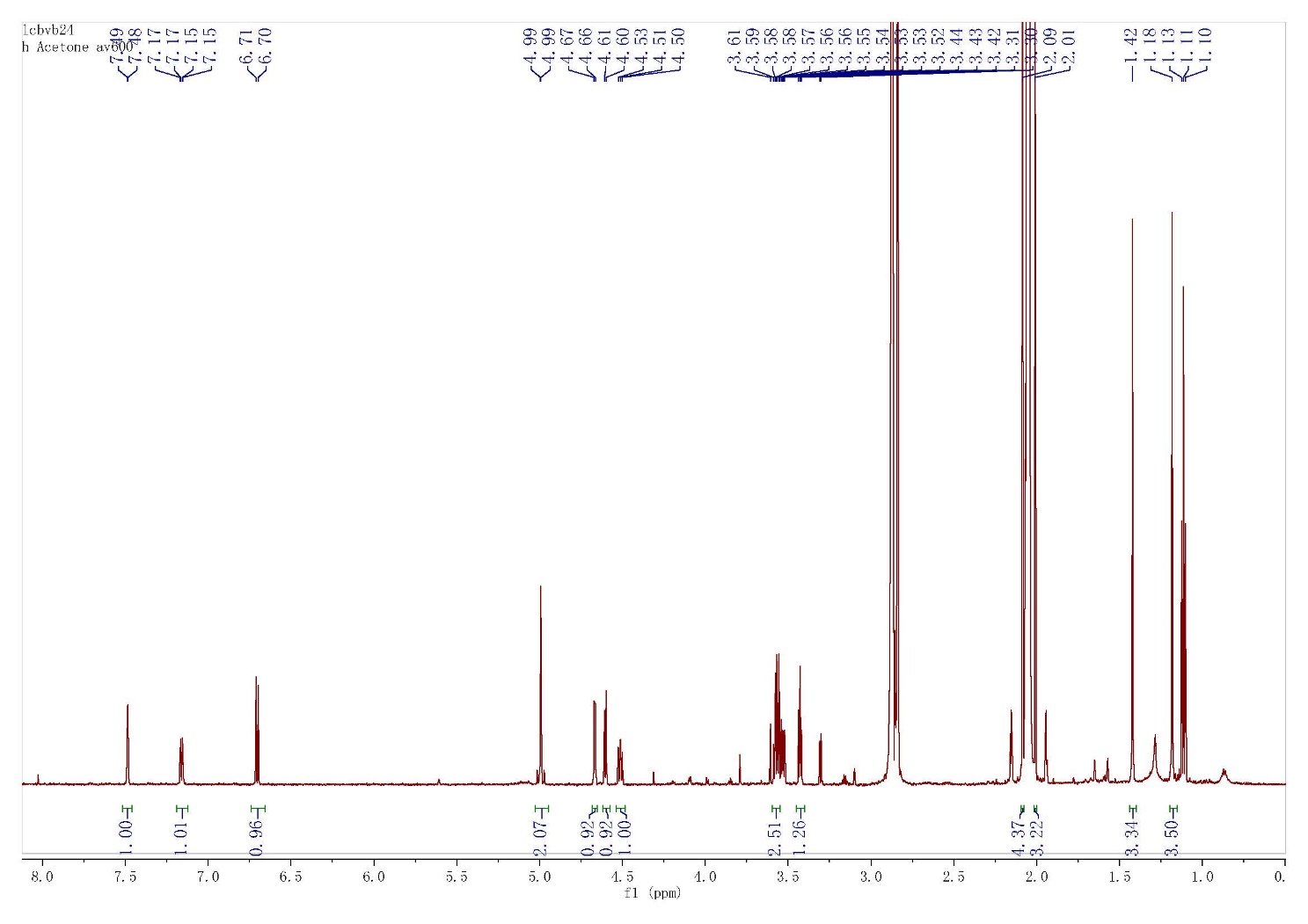


**Figure 30S**.^13^C NMR(200 MHz) and DEPT spectra of compound **5** in acetone-*d*_6_.


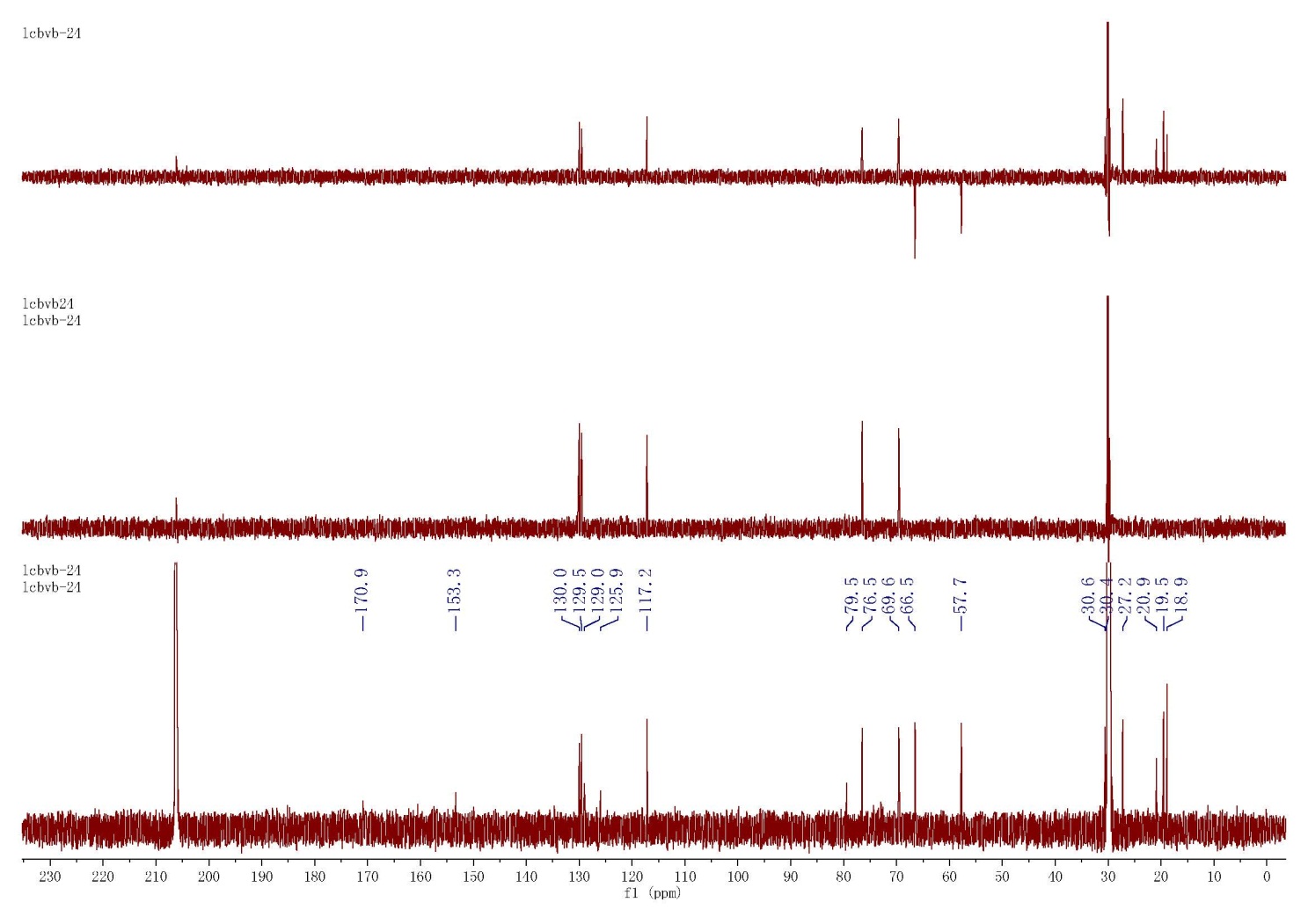


**Figure 31S.**HSQC (600 MHz) spectrum of compound **5** in acetone-*d*_6_.


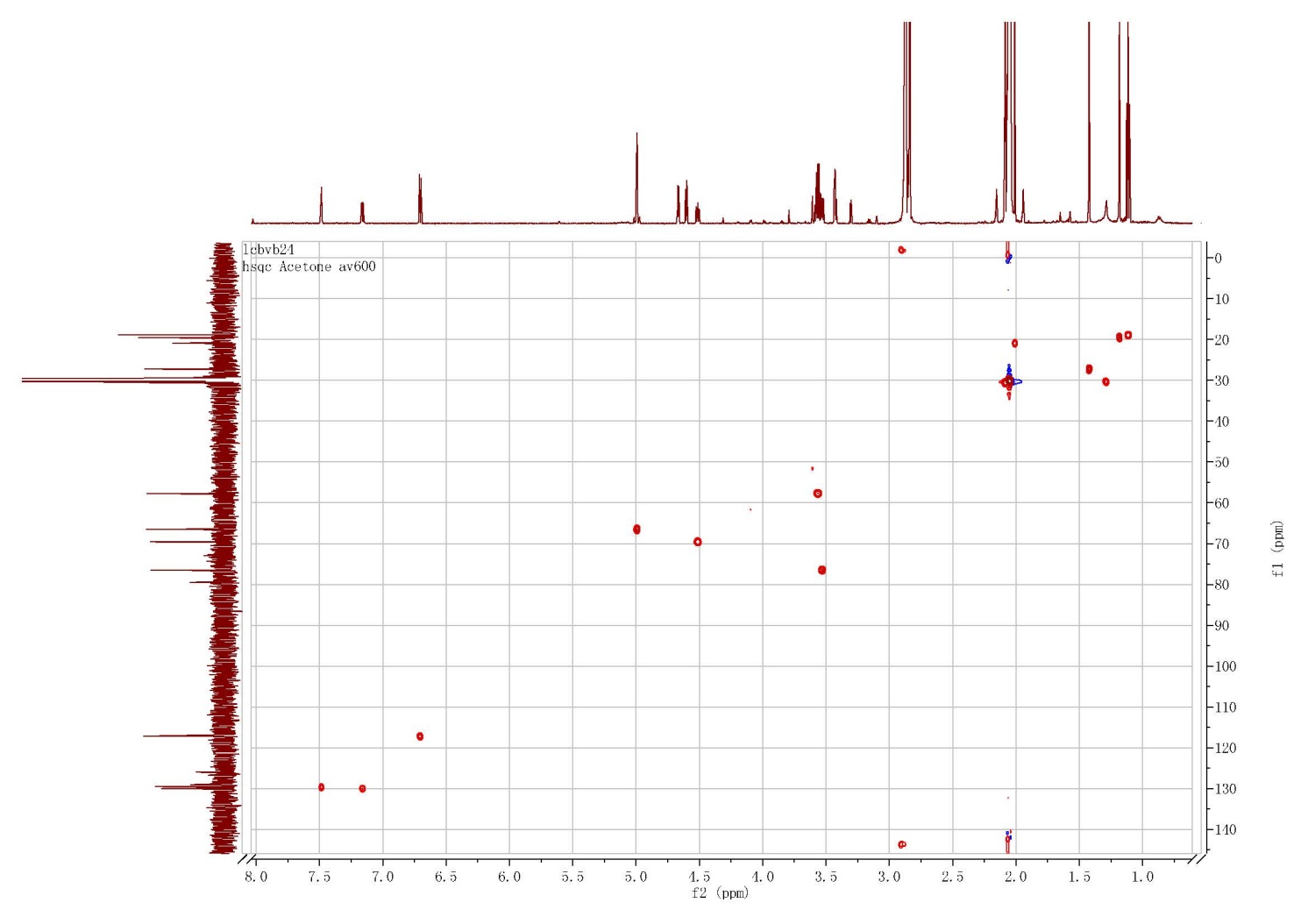


**Figure 32S.**HMBC (600 MHz) spectrum of compound **5** in acetone-*d*_6_.


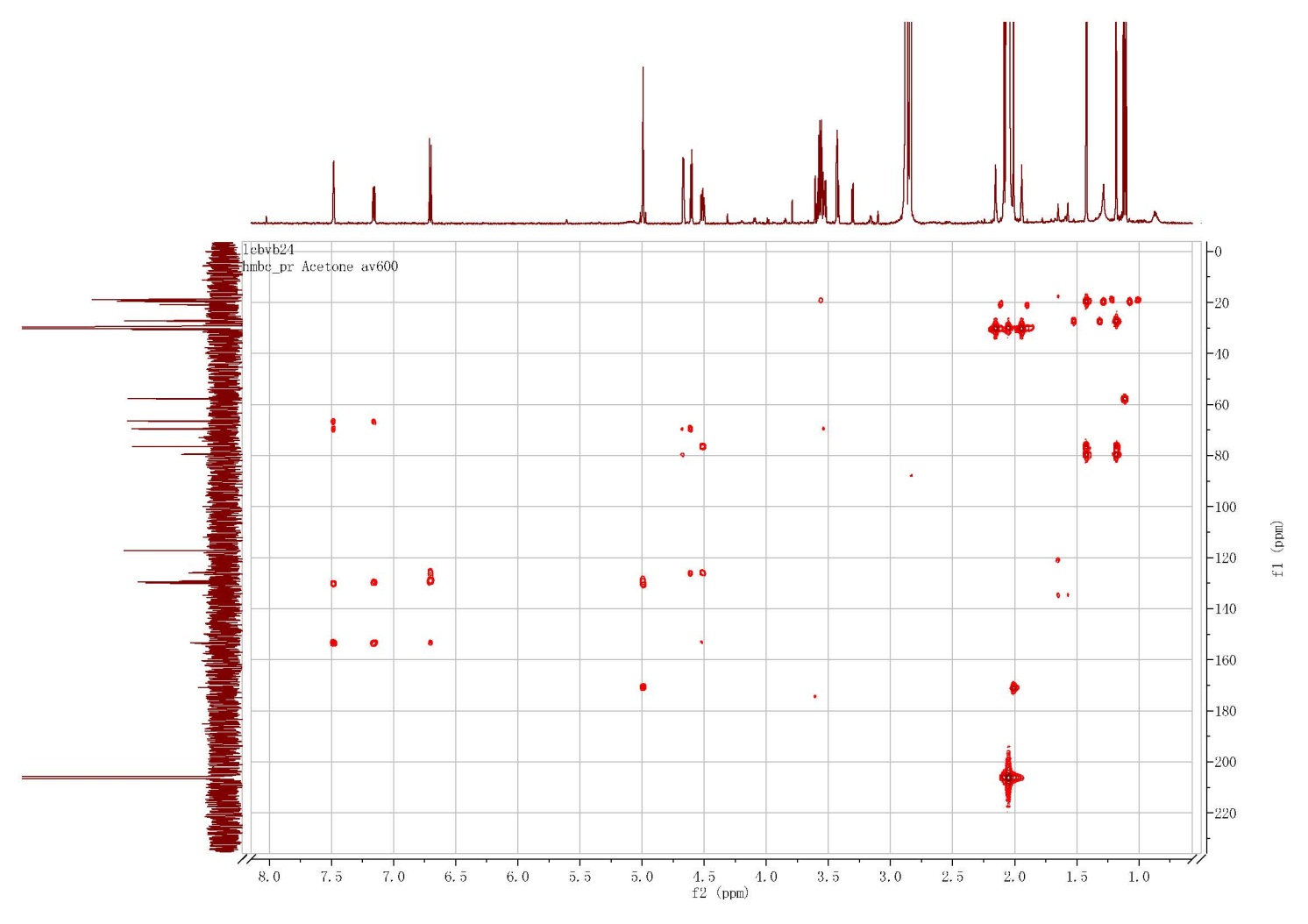


**Figure 33S.**^1^H-^1^H COSY (600 MHz) spectrum of compound **5** in acetone-*d*_6_.


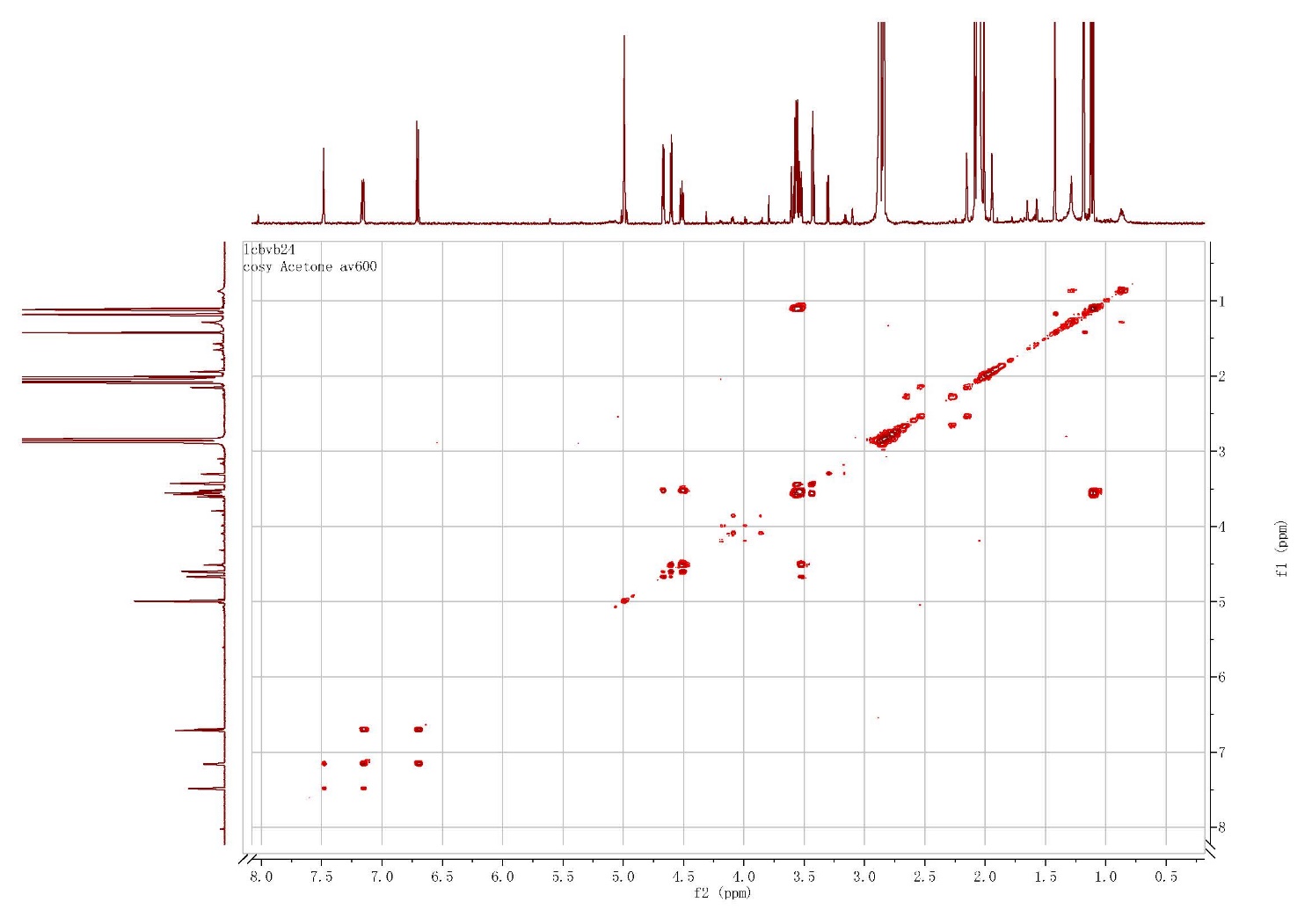


**Figure 34S.**ROESY (600 MHz) spectrum of compound **5** in acetone-*d*_6_.


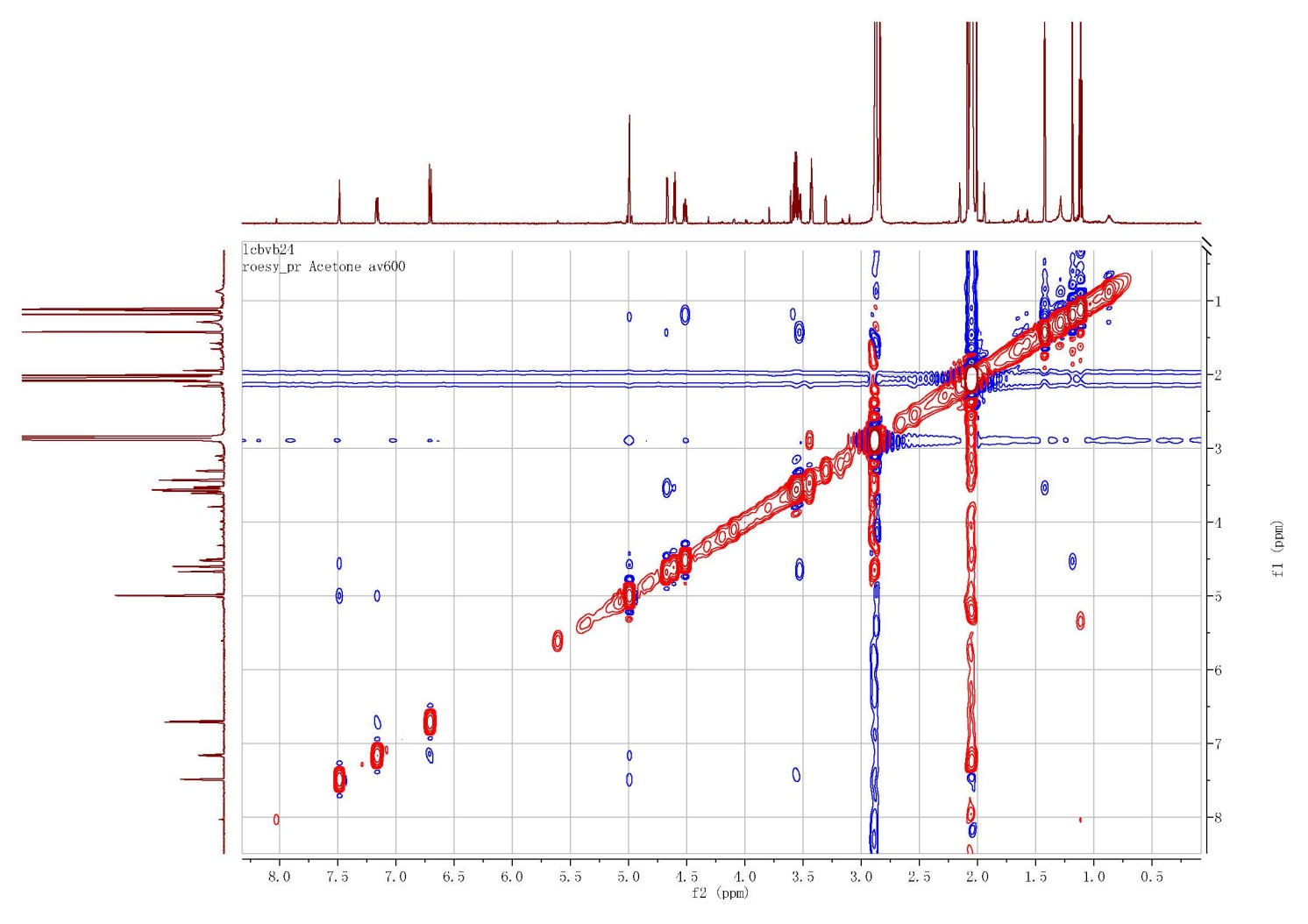


**Figure 35S.**HREIMS spectrum of compound **5**.


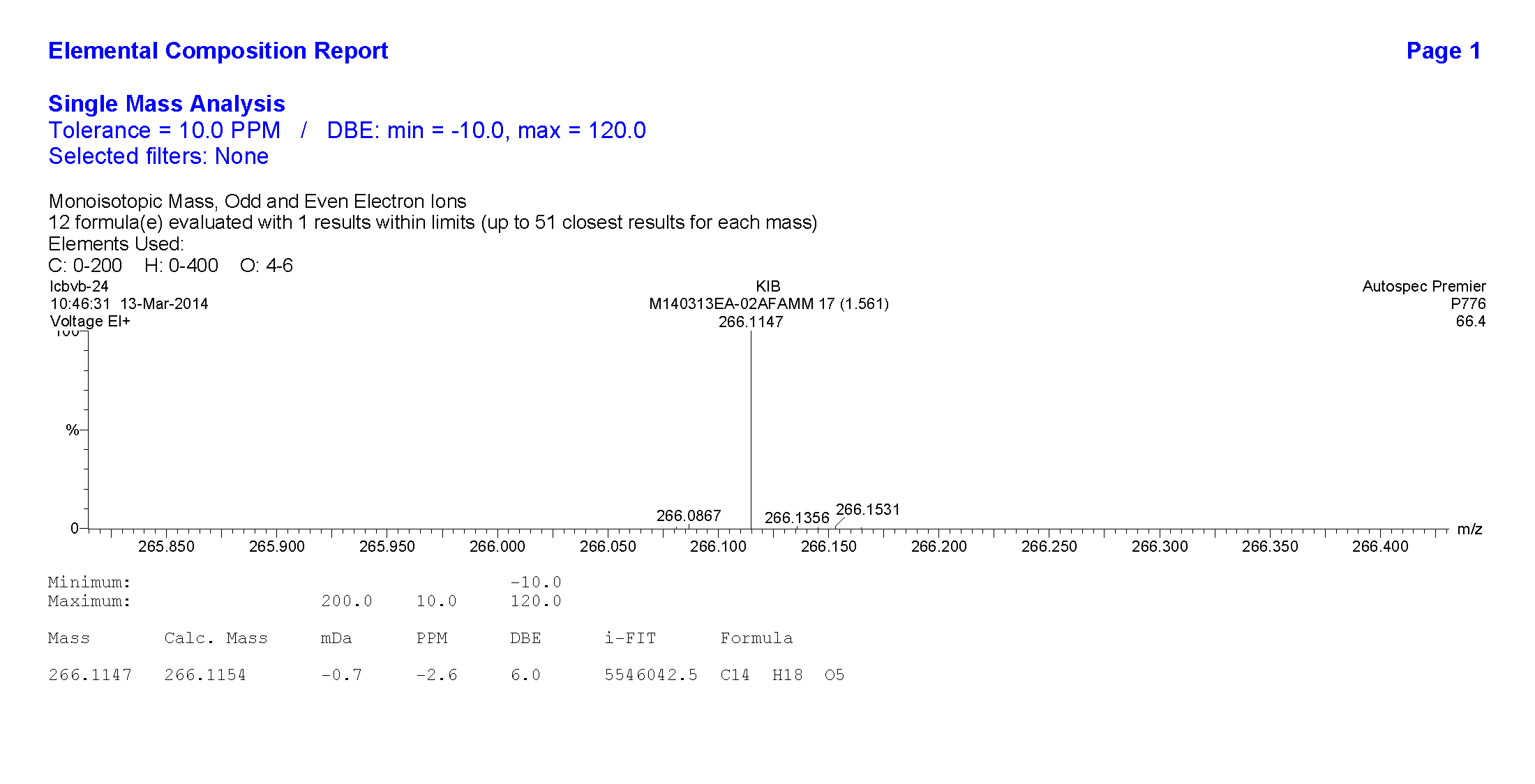


**Figure 36S**.^1^H NMR (600 MHz) spectrum of compound **6** in acetone-*d*_6_.


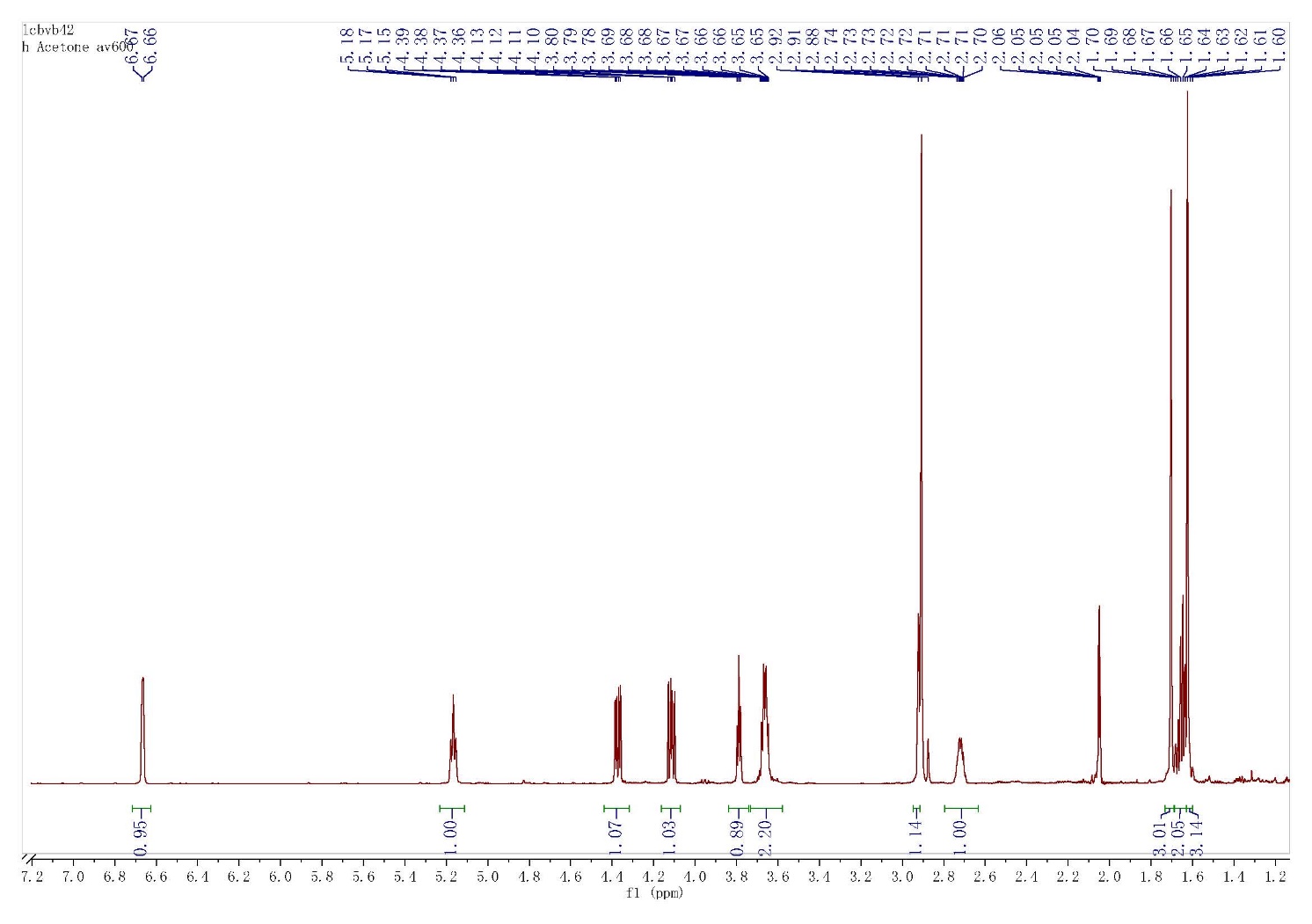


**Figure 37S**.^13^C NMR(150 MHz) and DEPT spectra of compound **6** in acetone-*d*_6_.


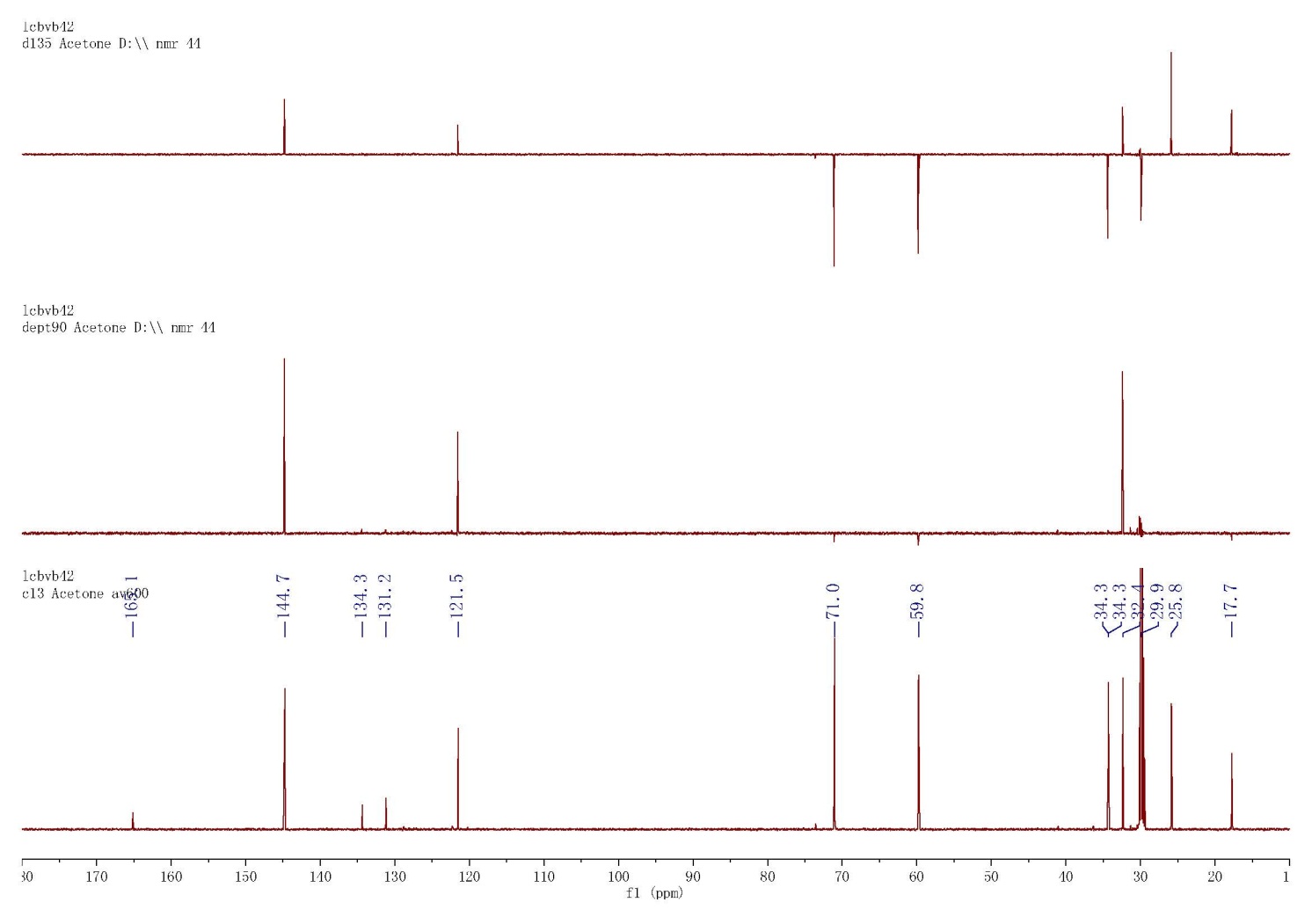


**Figure 38S.**HSQC (600 MHz) spectrum of compound **6** in acetone-*d*_6_.


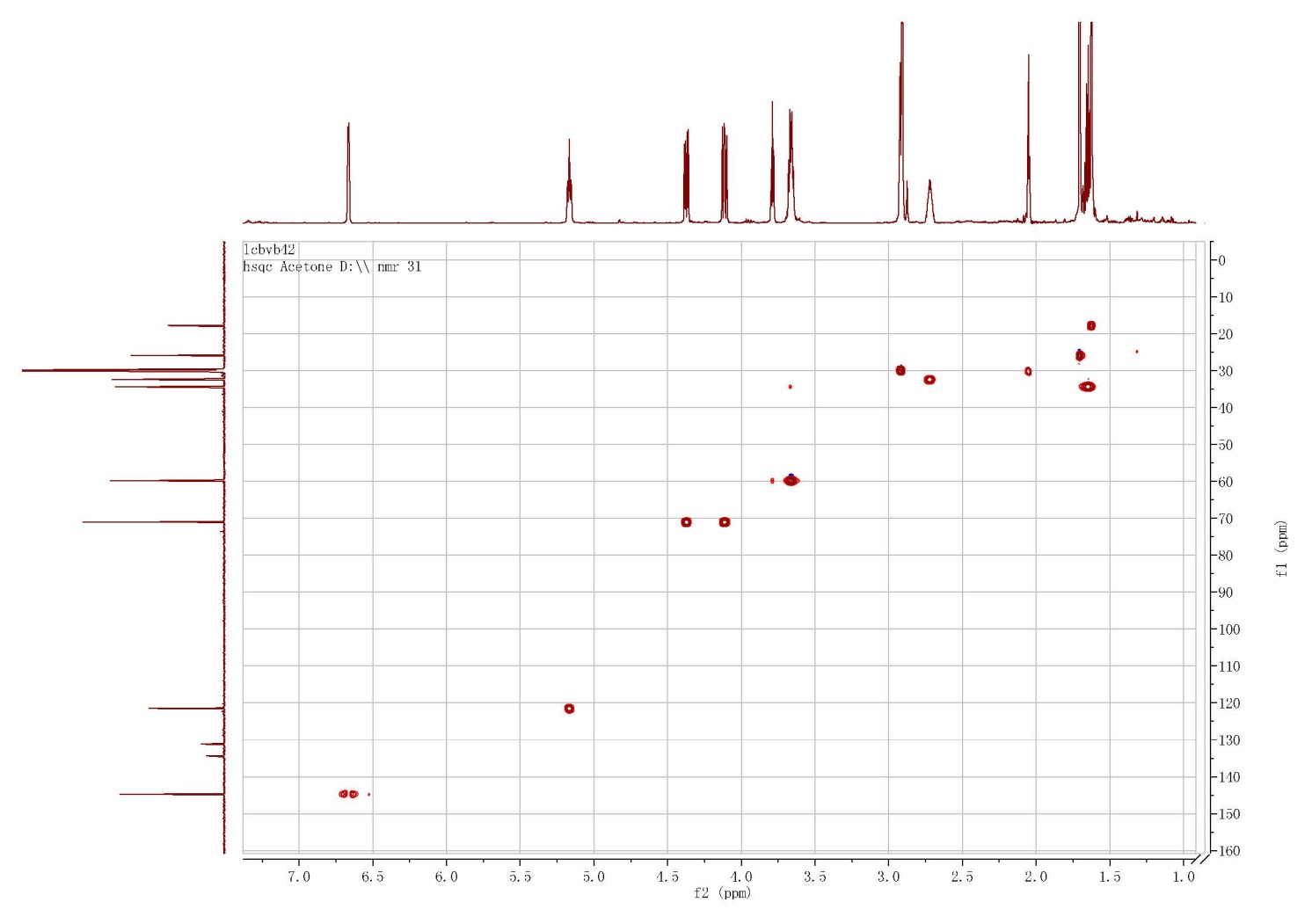


**Figure 39S.**HMBC (600 MHz) spectrum of compound **6** in acetone-*d*_6_.


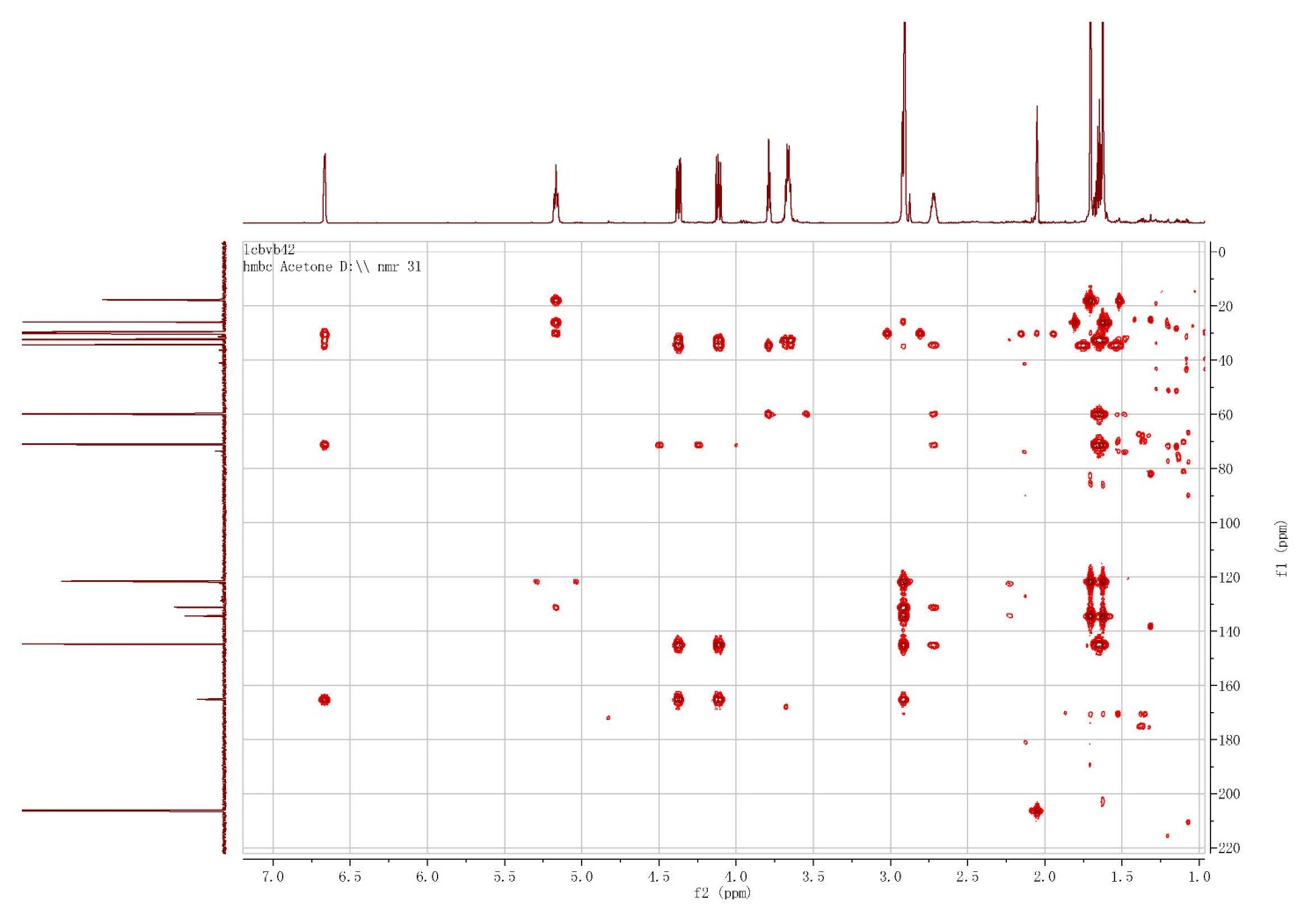


**Figure 40S.**^1^H-^1^H COSY (600 MHz) spectrum of compound **6** in acetone-*d*_6_.


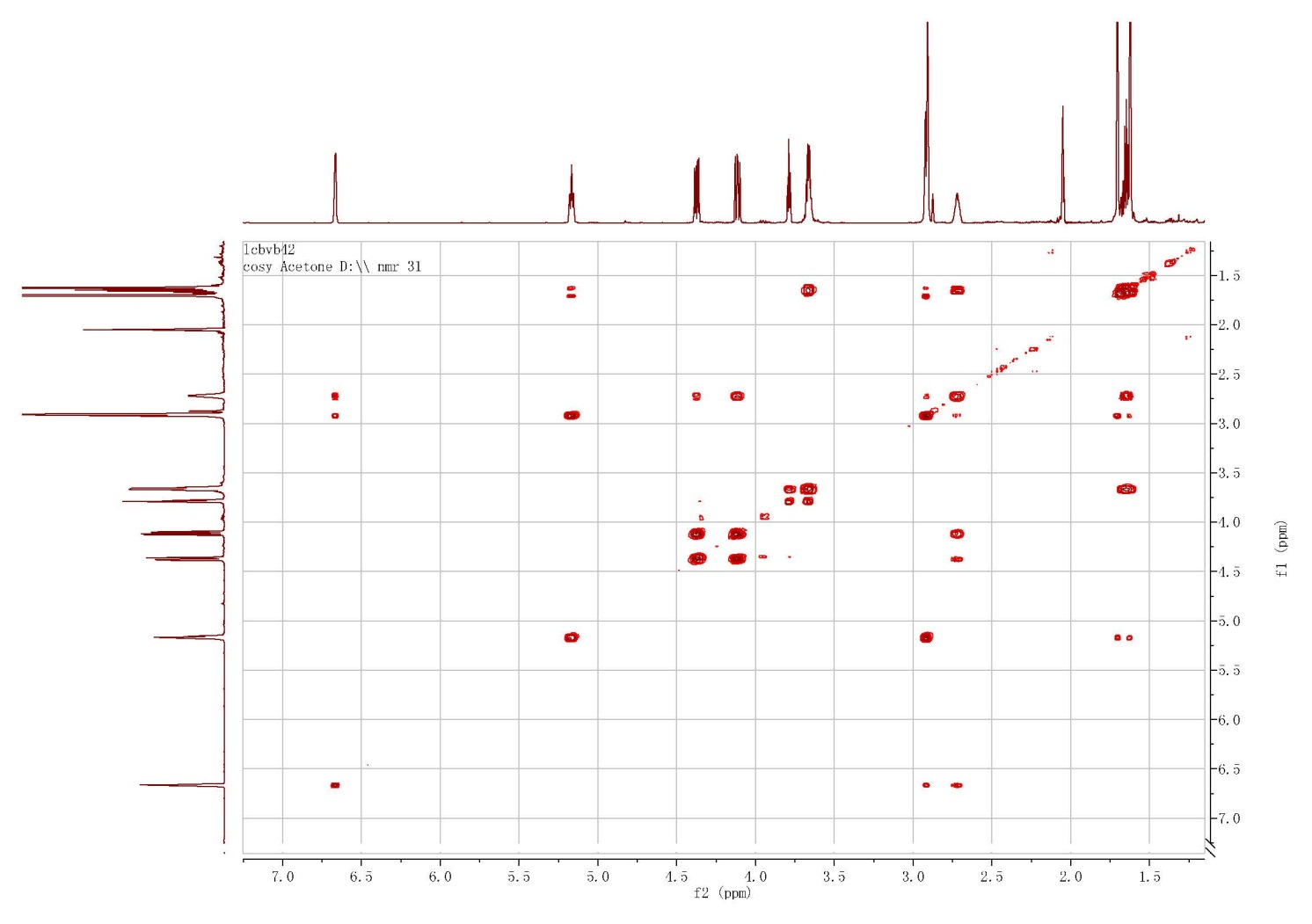


**Figure 41S.**ROESY (600 MHz) spectrum of compound **6** in acetone-*d*_6_.


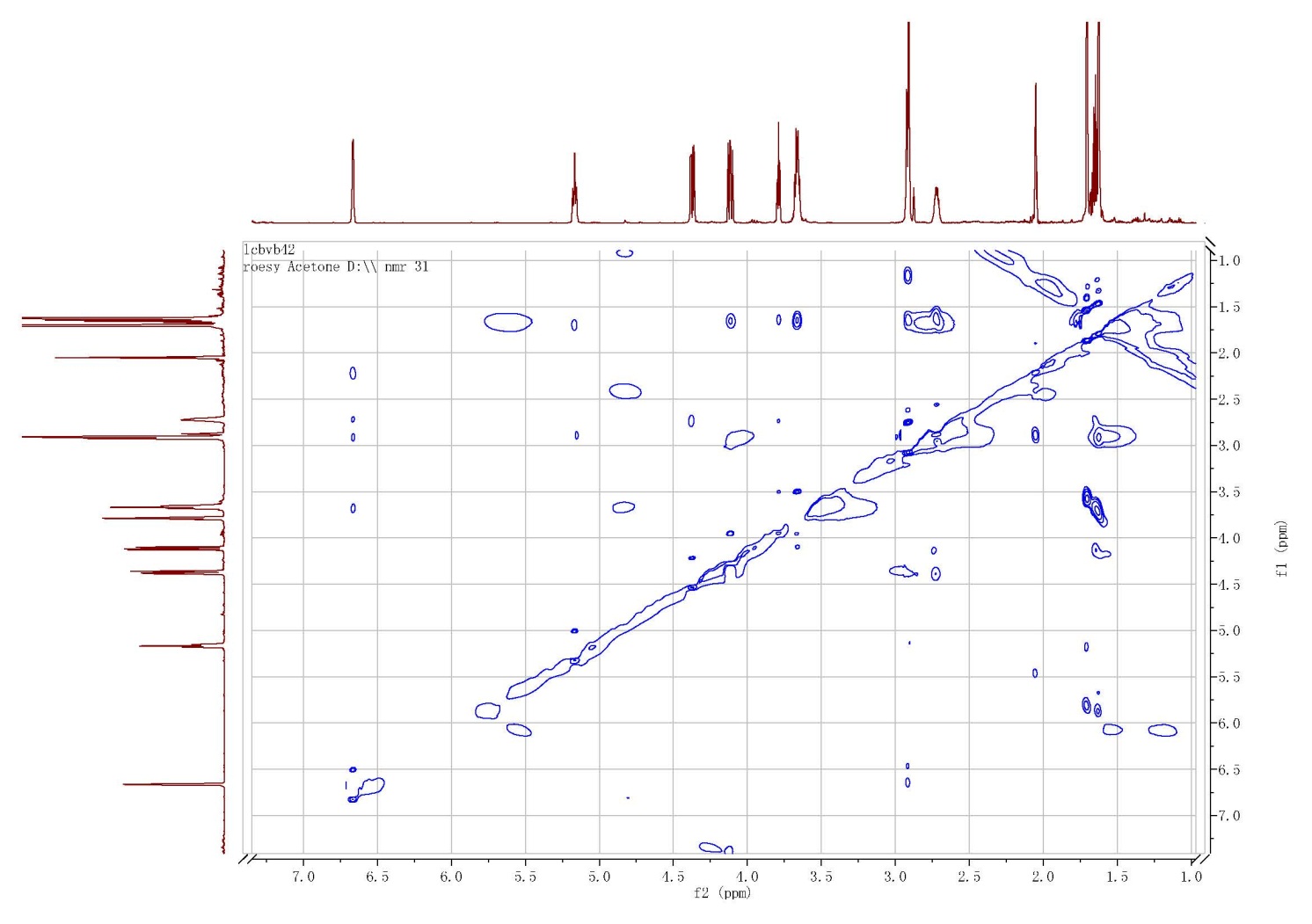


**Figure 42S.**HREIMS spectrum of compound **6**.


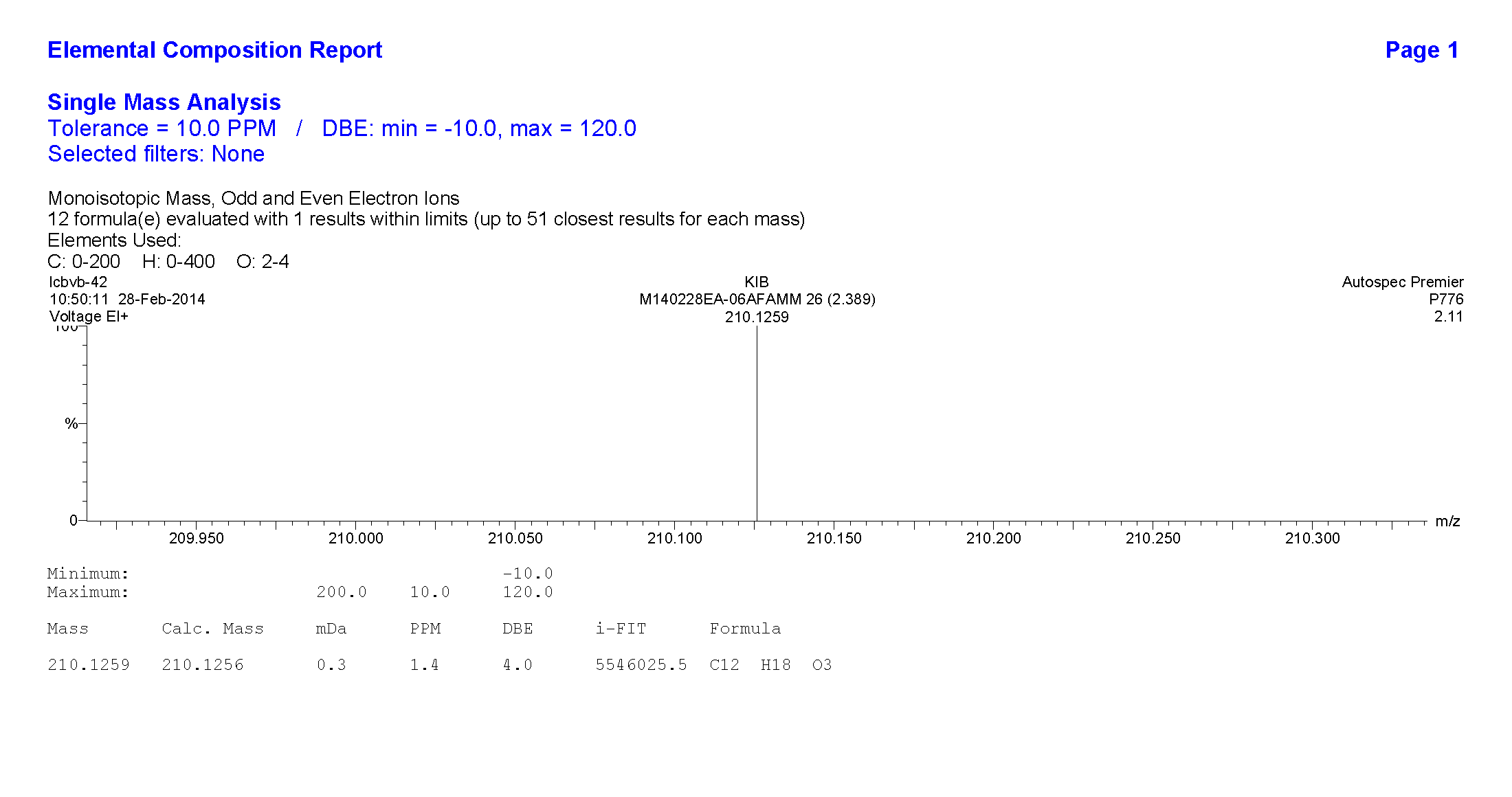

Supplement: Supplementary file 1 — Supplementary material 1 (DOCX 6801 kb) [file 13659_2014_29_MOESM1_ESM.docx]
